# New drugs and their performance 10 years after approval: a systematic analysis

**DOI:** 10.1007/s00210-025-04178-9

**Published:** 2025-05-08

**Authors:** Bores Manfouo, Roland Seifert

**Affiliations:** Institute of Pharmacology, Hannover Medical School, Hannover, D- 30625 Germany

**Keywords:** Pharmaceutical costs, AVR, Pharmaceutical sales, Drug prescriptions

## Abstract

More than 10,000 drugs are available on today’s market in Germany, with an increasing number receiving institutional approval every year. However, their general efficacy and utility for patients are often not thoroughly analyzed and, in some cases, remain questionable. The lack of systematic analysis for these parameters hinders learning from failures and successes. As a result, there is a risk of wasting resources by the pharmaceutical industry and approving new drugs that offer no additional benefit to patients in need of innovative treatments. Therefore, we set out to analyze the evolution of new drugs with innovative principles over 10 years after approval by the European Medicines Agency (EMA). We focused on drugs approved from 2004 to 2011, excluding protein kinase inhibitors and monoclonal antibodies, and identified 190 new drugs using the Arzneiverordnungsreport (AVR, Drug prescription report). With data from the Wissenschaftliches Institut der Ortskrankenkassen (WidO, Scientific Institute of the General Local Health Insurance Fund, AOK), we analyzed their number of prescriptions, sales, defined daily doses (DDD), and daily costs. We then extended our analysis with a focus on Rote-Hand-Briefe (RHB, Direct Healthcare Professional Communication). We identified factors of success, which was defined as a drug appearing in the top 3000. We then conducted a detailed analysis of outliers in terms of sales, focusing on parameters such as indications, innovation regarding their mechanism of action, relative costs, competition, pharmacological properties, and clinical studies. The analysis of both the most and least successful, allowed us to identify clear correlations and determine potential red flags as well as green flags regarding pharmaceutical sales. Nearly half (49%) of the drugs analyzed met our success criterion, most very early (66% within the first 2 years). Most of these drugs also showed a notable progression in the drug rankings over the years. Thirty percent of all analyzed drugs received RHBs, with most of them (84% of the said 30%) receiving at least one deemed potentially influential regarding sales. The successful drugs were more often subject to these potentially influential RHBs than their non-successful counterparts, and most of the potentially influential RHBs were related to adverse drug reactions (53%) or indications or contraindications (14.8%). Based on the analysis of the tops and flops, we conclude that market success, measured by sales, is influenced by multiple factors. These include indication(s), innovation, the competitive landscape, costs, and pharmacological aspects as well as studies regarding the efficacy and the adverse drug reactions of the drug. These results underline the necessity of a multifactorial approach based on value-adding to assess potential new drugs by pharmaceutical companies.

**Supplementary Information:**

The online version contains supplementary material available at 10.1007/s00210-025-04178-9.

## Introduction

More than 600 pharmaceutical companies are operating in Germany, generating a revenue of €59.8 billion € in 2023, with a growth rate of 5.4% compared to the previous year (GTAI, 2024, accessed October 18, 2024). On average, these companies invested approximately €5.7 billion (about $6.7 billion) in research and development (R&D) for each new drug in 2020 (Schuhmacher et al. 2023). While some new drugs achieve “blockbuster” status, delivering significant health benefits for patients and economic returns for pharmaceutical companies, many perform poorly in the market. Such underperformance may result from a lack of innovation (Stafford 2014) or an absence of added value for patients, often compounded by exorbitant and arbitrary pricing strategies. These factors contribute to escalating health expenditures, posing risks to the economic stability of healthcare systems (Hofbauer-Milan et al. 2023; Haserück et al. 2022). Within this context, it is increasingly critical to maximize the value derived from every € spent. The objective of this study was to identify newly approved drugs that lack sufficient added value, with the aim of learning from past mistakes and proposing solutions that can lead to better drugs being approved in the future. This would ensure that R&D investments by pharmaceutical companies are allocated effectively, ultimately benefiting patient care. The Arzneiverordnungsreport (AVR) serves as a key resource to that objective by providing detailed data and analysis on prescription patterns and drug usage within the German healthcare system. It includes information on prescribing frequencies, cost trends, and variations in prescription practices, helping inform healthcare decisions and policymaking. Among other topics, it presents new drugs with innovative principles released during the year, including information such as the context of approval, efficacy, and an initial recommendation regarding their potential use in patient treatment.

## Materials and methods

**Fig. 1:**
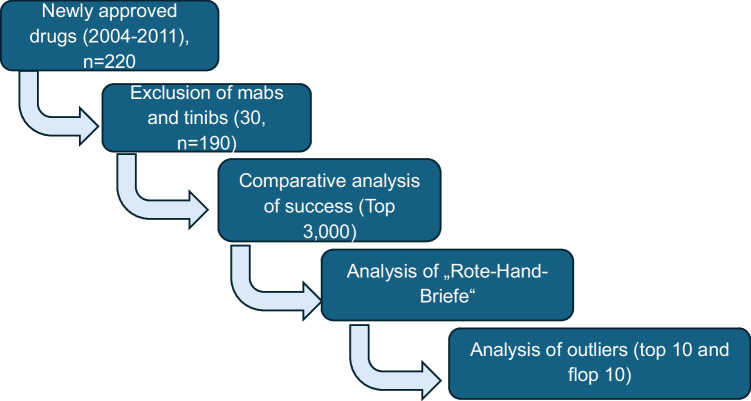
Flowchart of the analytical procedure

Figure 1 gives an overview of the systematic analysis we performed in terms of drug selection:

### General analysis

Our data collection was primarily based on the AVR. We identified newly introduced drugs with innovative mechanisms of action that became available between 2004 and 2011 (Schwabe and Paffrath 2005; Schwabe and Paffrath 2006; Schwabe and Paffrath 2007; Schwabe and Paffrath 2008; Schwabe and Paffrath 2009; Schwabe and Paffrath 2010; Schwabe and Paffrath 2011; Schwabe and Paffrath 2012). Subsequently, we excluded monoclonal antibodies and tyrosine kinase inhibitors, resulting in a final cohort of 190 drugs. This exclusion was due to the fact that these “targeted therapeutics” with their particular characteristics, such as a relatively narrow indication spectrum as oncological drugs for most of them, and their pharmacoeconomic characteristics warrant a more thorough analysis taking a deeper look at these parameters. Such analyses are already taking place in our institute, and some contributions are already available concerning tyrosine kinase inhibitors (Obst and Seifert 2023; Obst and Seifert 2025), with analyses for monoclonal antibodies ongoing.

### Top 3000 comparative analysis

We identified drugs included in the annual “successful” list of the 3000 most prescribed medications of the AVR and tracked their performance over 11 years, from the year of approval up to 10 years after the approval. Additional data were obtained from the 2013 to 2020 editions of the AVR (Schwabe and Paffrath 2013; Schwabe and Paffrath 2014; Schwabe and Paffrath 2015; Schwabe and Paffrath 2016; Schwabe et al., 2017; Schwabe et al. 2018; Schwabe et al. 2019; Schwabe and Ludwig 2020) as well as from the online database PharMaAnalyst provided by WidO (https://arzneimittel.wido.de/PharMaAnalyst/, accessed September 10, 2024). We analyzed the drugs’ performance metrics, including their first year of success, the duration of their success, and their progression within the top list.

### “Rote-Hand-Briefe,” direct healthcare professional communication

Following the initial analysis, we proceeded to examine the “Rote-Hand-Briefe” (RHBs) pertinent to our drugs cohort. RHBs are official communications issued by pharmaceutical companies or regulatory authorities to alert healthcare professionals about significant safety information related to specific drugs. We reviewed all RHBs from the BfArM (Bundesinstitut für Arzneimittel und Medizinprodukte, “Federal Institute for Drugs and Medical Devices”) and AkdAe (Arzneimittelkommission der deutschen Ärzteschaft, “Drug Commission of the German Medical Association”) databases that were issued during the analyzed period for the drugs in our cohort (BfArM: https://www.bfarm.de/DE/Arzneimittel/Pharmakovigilanz/Risikoinformationen/Rote-Hand-Briefe/_node.html, accessed September 10, 2024; akdae: https://www.akdae.de/arzneimittelsicherheit/rote-hand-briefe, accessed September 10, 2024). We developed a classification system to categorize these RHBs based on their content and potential impact (positive, negative, or neutral) on the number of prescriptions for the respective drugs (Fig. 1). The data were subsequently analyzed with a focus on comparing the prevalence and types of RHBs issued for successful drugs versus their non-successful counterparts (Table 1).

**Table 1:** Classification of “Rote -Hand Briefe”

RHB categories (sales related)	
Potentially influential	Potentially negligible
Reminders—limitations to the indications	Technical issues
Contraindications	Instructions to correct use**—**potentially negligible
Instructions for correct use—potentially influential	
New adverse drug reactions or concerns	
Higher mortality or lethal adverse drug reactions	
Partial market withdrawal	
Complete market withdrawal	

### Tops and flops

Additional and more precise data regarding prescriptions and sales (measured in defined daily doses (DDDs)) for each analyzed drug were obtained from WidO (https://www.wido.de/, accessed September 10, 2024). We identified the 10 most successful (top 10) and the 10 least successful drugs (flop 10) based on the number of DDDs. DDDs data provided by WidO reflect drugs used in outpatient settings that are accessible via prescriptions. Consequently, drugs used in other settings (such as in-hospital treatments, chemotherapy, or dialysis units) that are not available through prescriptions (i.e., not sold in pharmacies opened to the public) may be underrepresented. Based on that, certain drugs, particularly those originally classified as “flops,” were excluded from the analysis. Additionally, orphan drugs were excluded from the “flops” category due to their inherently lower sales volumes compared to other drugs. A detailed analysis of the highest- and lowest-performing drugs ensued, with the additional help of the online databases arznei-telegramm (https://www.arznei-telegramm.de/01index.php3, accessed October 18, 2024) and pharmazeutische Zeitung (https://www.pharmazeutische-zeitung.de/, pharmaceutical magazine, accessed October 18, 2024) examining various factors such as indications, mechanism of action, pricing, competitive landscape, pharmaceutical characteristics, and patient outcomes observed in studies conducted before EMA approval.

To evaluate the market coverage of specific drugs, calculations were based on the following formula:

$$DID\;prescriptions=\frac{DDD\;prescriptions}{population\times\;356}\times\;1000$$

Next, treatment coverage per indication was determined to assess the relationship between the use of a specific drug and the prevalence of the corresponding medical indications. This measure reflects the percentage of patients with a given indication who are receiving treatment with the specified drug.

Treatment coverage was calculated using the formula:

$$Treatment\;coverage\;in\;\%=\;\frac{DID\;prescriptions}{prevalence\;in\;\%\;\times\;10}$$

## Results and discussion

### General analysis

**Fig. 2:**
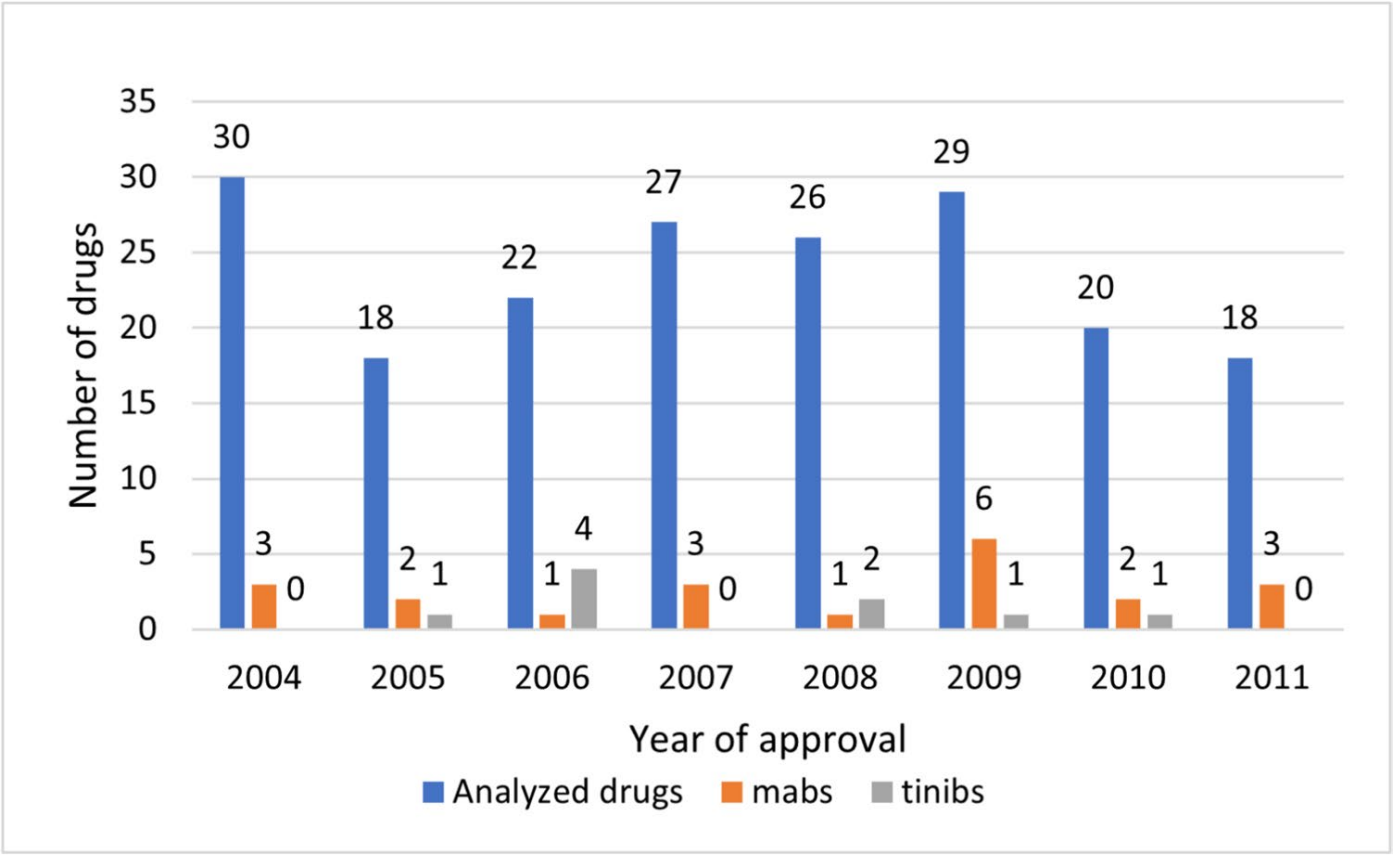
General statistics for new drugs per year (2004–2011)

We identified a total of 190 drugs over 8 years, for a median of 26 drugs approved per year and a range from 18 (2005) to 30 (2004). These 190 drugs constitute the majority (86.36%) of all drugs approved during the analyzed period. Monoclonal antibodies (9.55%) and tyrosine kinase inhibitors (4.09%) accounted for relatively small proportions of the total number of drugs (Fig. 2).

### TOP 3000

**Fig. 3:**
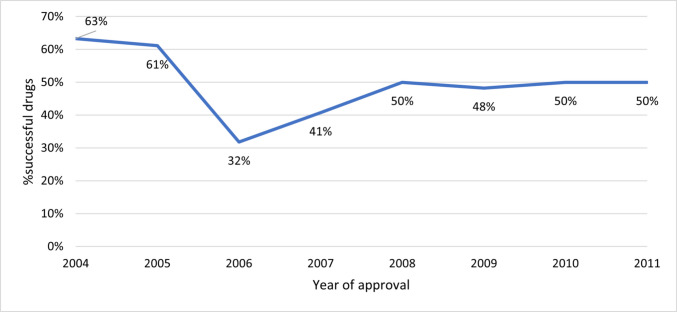
Success rates of drugs by year of approval (2004–2011)

**Fig. 4:**
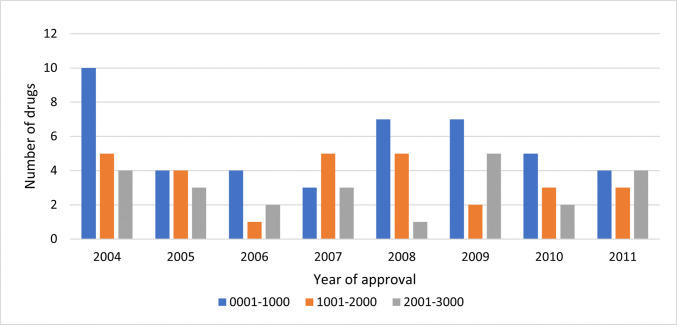
Peak performance for the successful drugs within the first 10 years post-approval, peak performance being defined as the highest ranking achieved by a drug in the analyzed period

In terms of market success, 49% of the analyzed drugs met our criterion by appearing on the top 3000 list at least once. Notably, only 31.82% of the drugs from 2006 met this criterion (Fig. 3). Among these successful drugs, 46% advanced further to the top 1000 list (Fig. 4).

By combining the relatively modest success criterion (top 3000) with the observation that more than half of the analyzed drugs could not meet that criterion, it becomes clear that a significant number of drugs may not add substantial value to patient care. However, some of these drugs may require additional evaluative metrics, beyond sales figures, to be considered a failure. This includes medications targeting orphan diseases or those primarily utilized in inpatient settings. This observation holds for many drugs approved from 2011 to 2017, where an added benefit was found in only 30% of the analyzed drugs by the GBA (“Gemeinsamer Bundesausschuss,” Federal Joint Commission) in early benefit assessments (Peinemann and Labeit 2019).

**Fig. 5:**
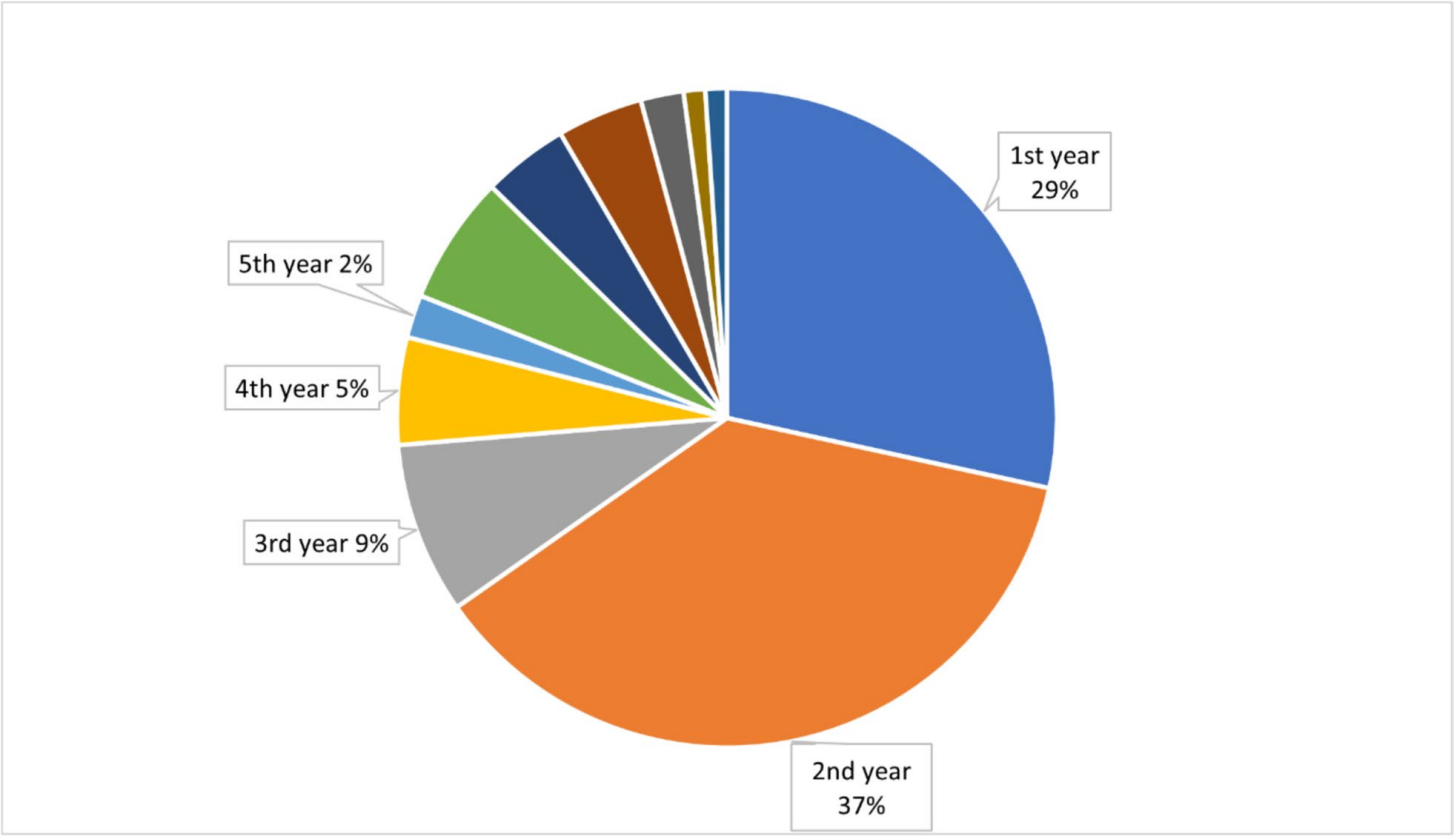
Year of first market success

Most of the successful drugs (66%) achieved this status within their first 2 years on the market, as we noticed a clear decline in the likelihood of success with time. For instance, only approximately 9% of the initial successes occurred in the third year, 5% in the fourth year, and 2% in the fifth year (Fig. 5).

**Fig. 6:**
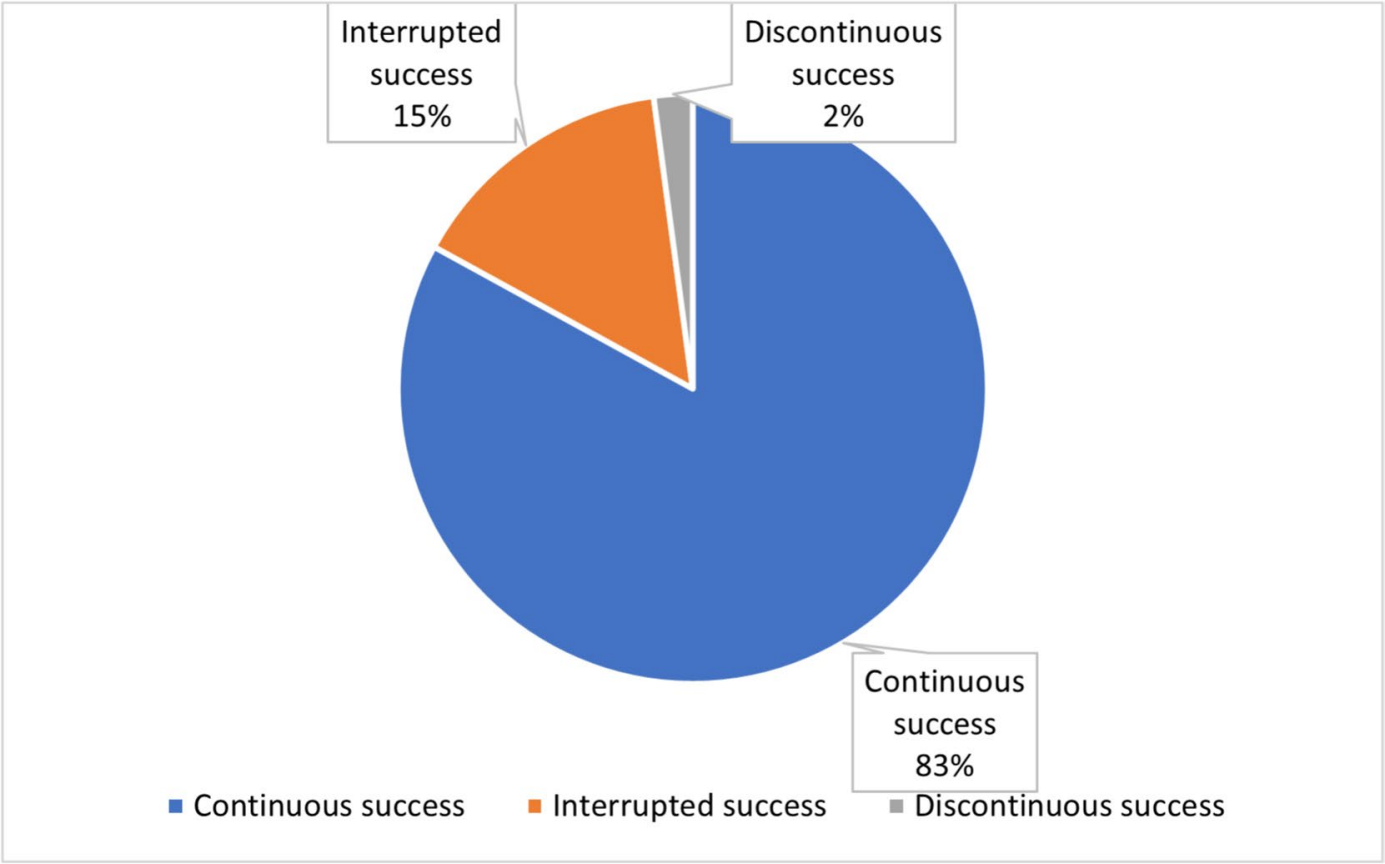
Continuity of performance (remaining in the top 3000)

**Fig. 7:**
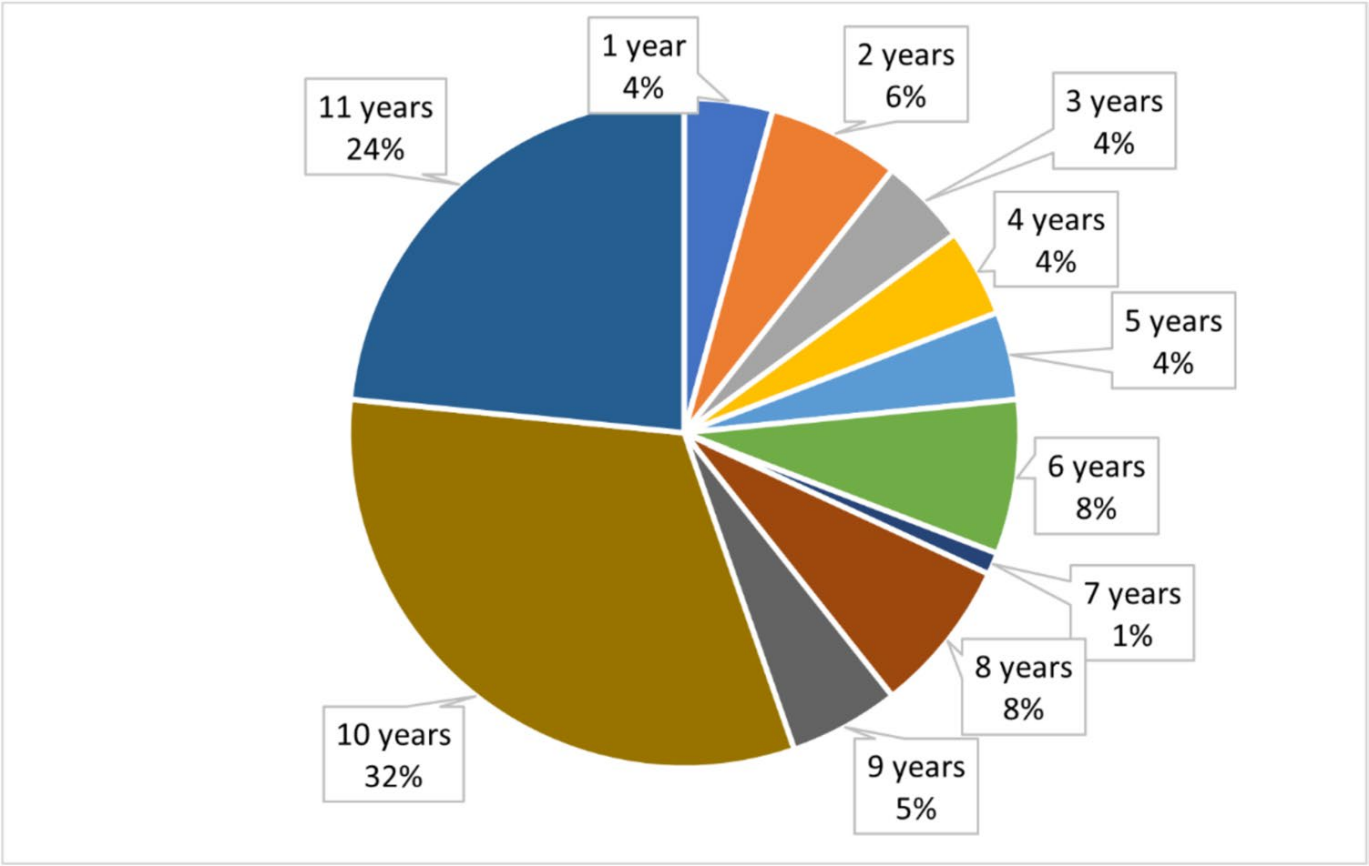
Duration of success, staying in the top 3000

Most successful drugs (83%) remained in the top 3000 list throughout the analyzed period. The early success combined with consistent performance explains why around 61% of the successful drugs remained on the top list for at least 9 out of 11 years (Figs. 6 and 7). We defined “discontinuous success” as leaving the top 3000 list temporarily and re-entering it some years later, and “interrupted success” as leaving the top 3000 without re-entering.

**Fig 8:**
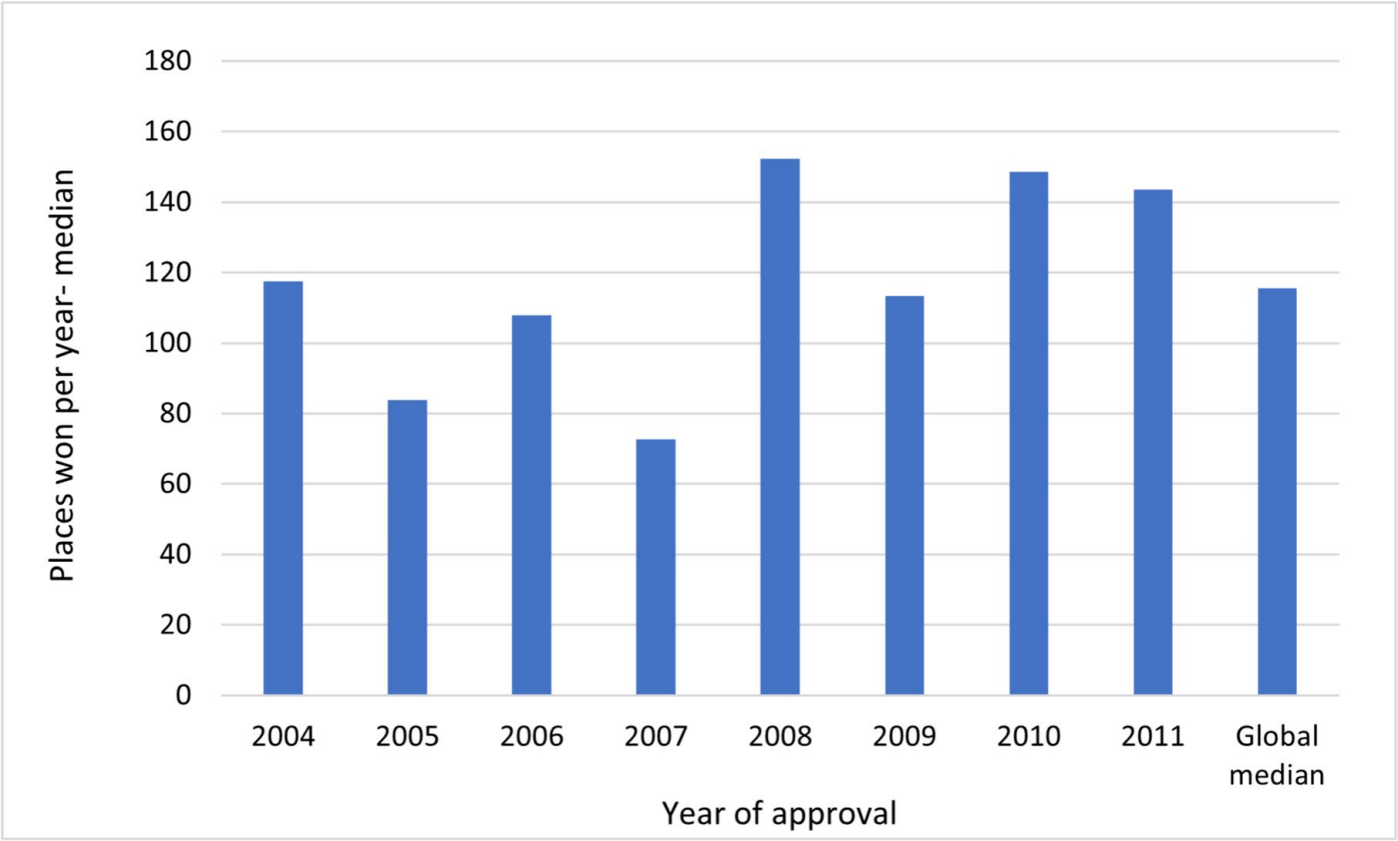
Median rank progression of the successful drugs

The progression of successful drugs also demonstrated a steady upward trend. On average, these drugs advanced nearly 126 positions in the ranking each year, with a range from approximately 84 positions for drugs released in 2005 to 194 positions for the 2011 class (Fig. 8).

### RHBs

Approximately 30% of the analyzed drugs were subject to RHBs, with the proportion ranging from 15% in 2010 to 44.4% in 2011 (Fig. S1 in the supplement).

**Fig. 9:**
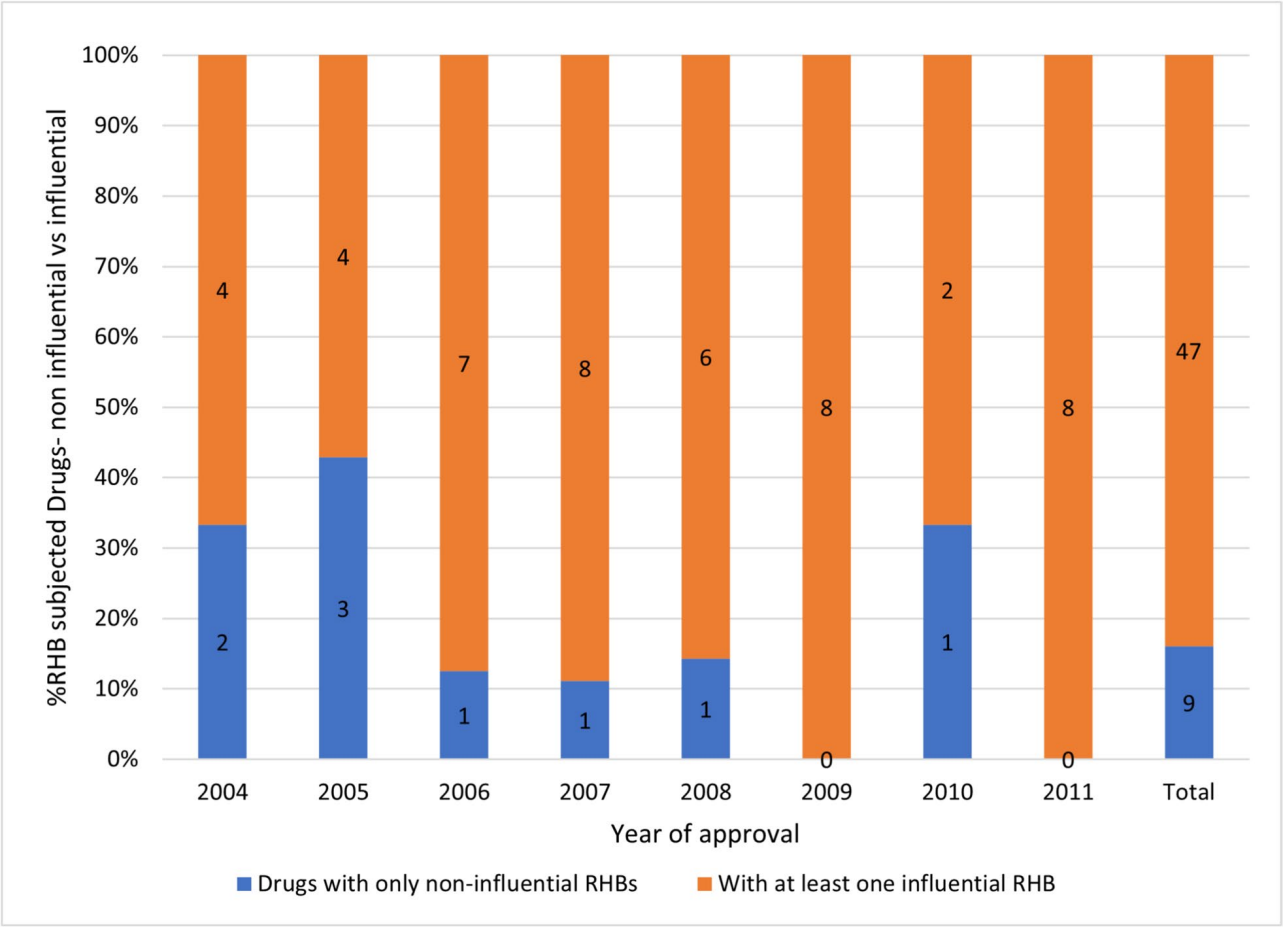
Comparison of drugs subject to potentially influential vs potentially negligible RHBs (Raw counts by category in bars)

**Fig. 10:**
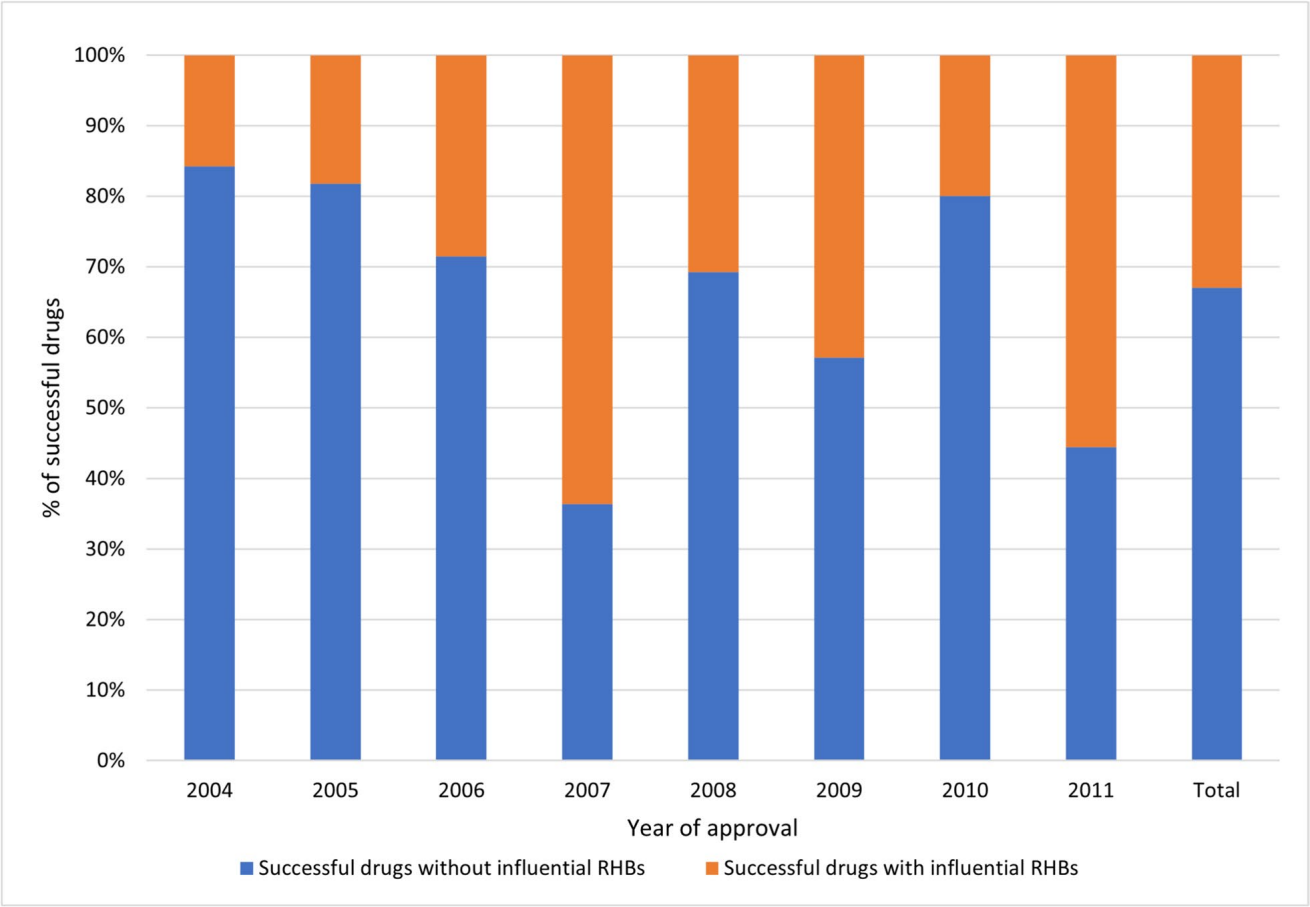
Successful drugs and potentially influential RHBs

**Fig. 11:**
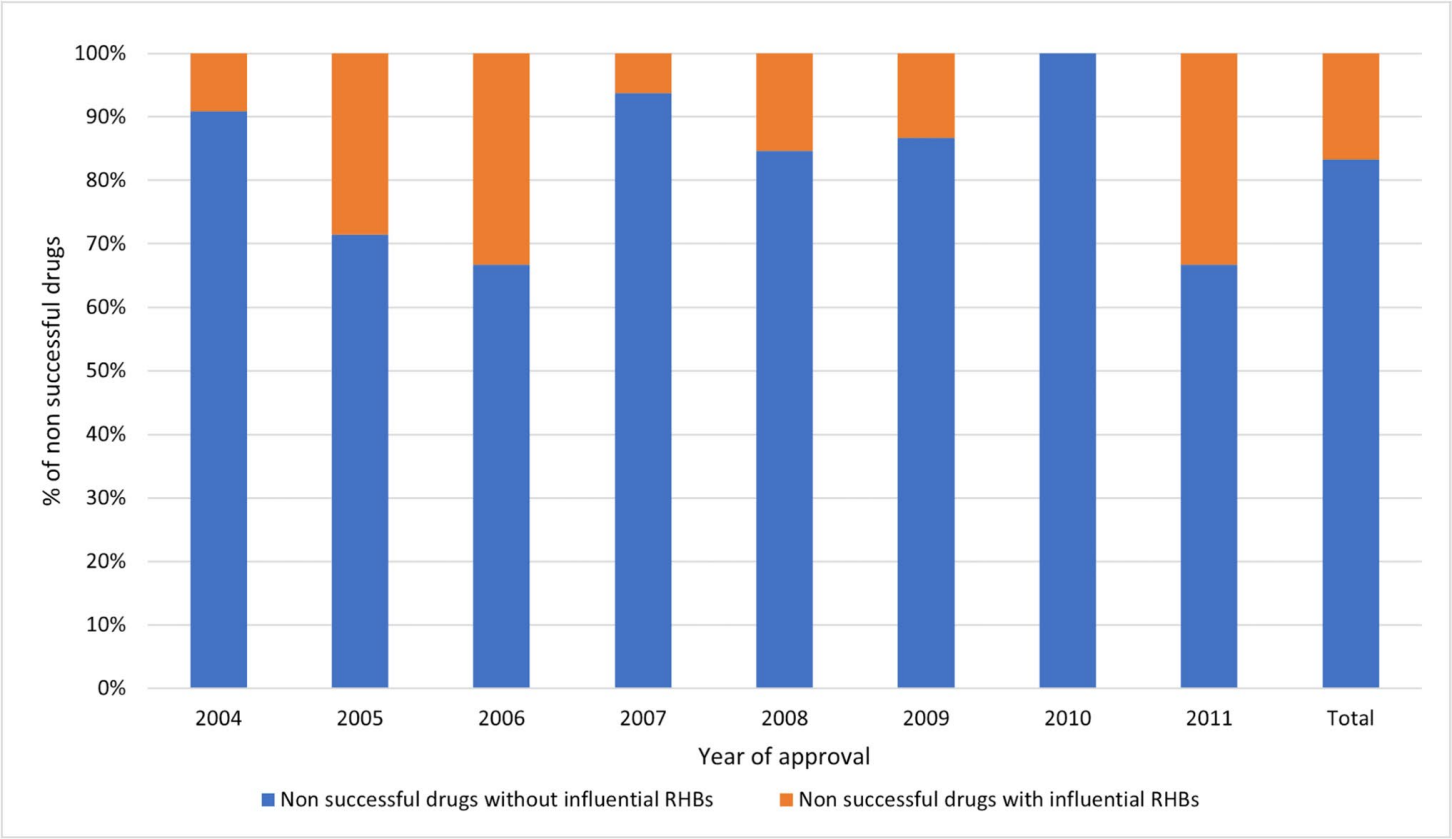
Non-successful drugs and potentially influential RHBs

Around 84% of the RHB-subjected drugs received at least one potentially influential RHB, with proportions ranging from 57% in 2005 to 100% in 2009 and 2011 (Fig. 9). Comparative analysis revealed that successful drugs were more often subject to potentially influential RHBs than their non-successful counterparts. Specifically, 33% of the top 3000 drugs were subject to potentially influential RHBs, with a range from 16 to 64%, against 16.7% for the non-successful drugs, with a range from 0 to 66.7% (Figs. 10 and 11). The chi-square analysis revealed a significant association between drug success and the receipt RHBs, with an odds ratio (OR) of 2.46 (95% CI [2.24, 4.89], *p* < 0.05). To further examine this correlation, more carefully planned studies with adequate study designs are needed. Such studies would contribute to deepening our understanding of the effect RHBs can have on drug success.

Given that potentially influential RHBs are more prevalent among successful drugs, we argue that the mere presence of RHBs should not be regarded as a universal red flag in the context of drug sales. Rather, this association could be interpreted positively, as it suggests that successful drugs are utilized more frequently, thereby increasing the likelihood of identifying previously undetected adverse drug reactions and their potential consequences.

Overall, we find potentially influential RHBs to be a relatively poor indicator of bad-performing drugs in terms of sales, since their presence does not seem to indicate lower drug sales. Regarding their impact on prescription patterns, previous studies from countries such as Australia, Canada and Denmark have reported an average reduction of approximately 6% following the issuance of a safety warning, similar in concept to RHBs (Mintzes et al. 2022). This moderate effect can be attributed to several factors: healthcare professionals (HCPs) tend to place greater trust in communications from regulatory authorities and medical associations than those from the pharmaceutical industry. Additionally, many RHBs are suboptimal in design, rendering them challenging for HCPs to interpret and act upon effectively (Møllebæk et al. 2019).

**Fig. 12:**
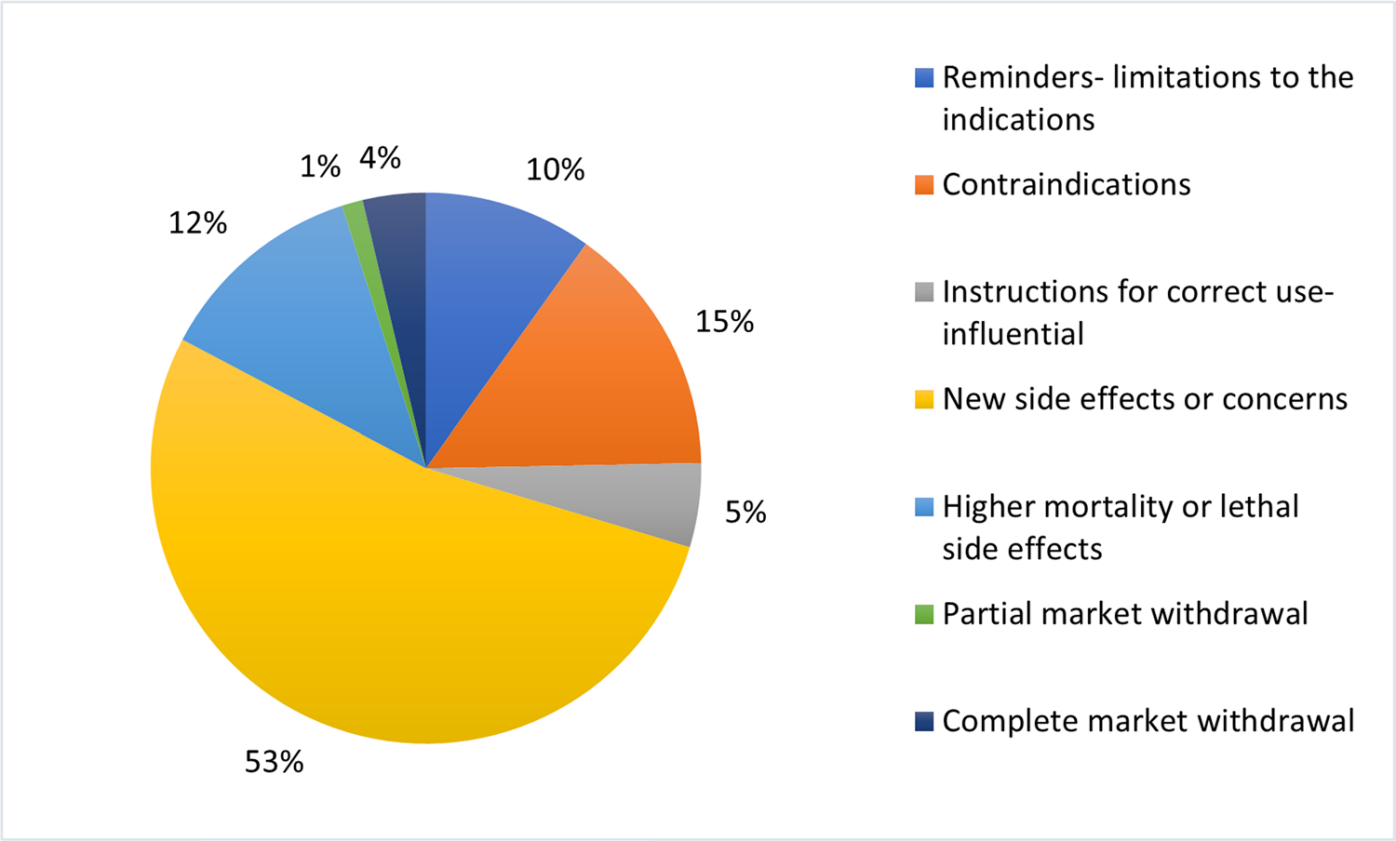
Content of potentially influential RHBs for the analyzed contingent

**Fig. 13:**
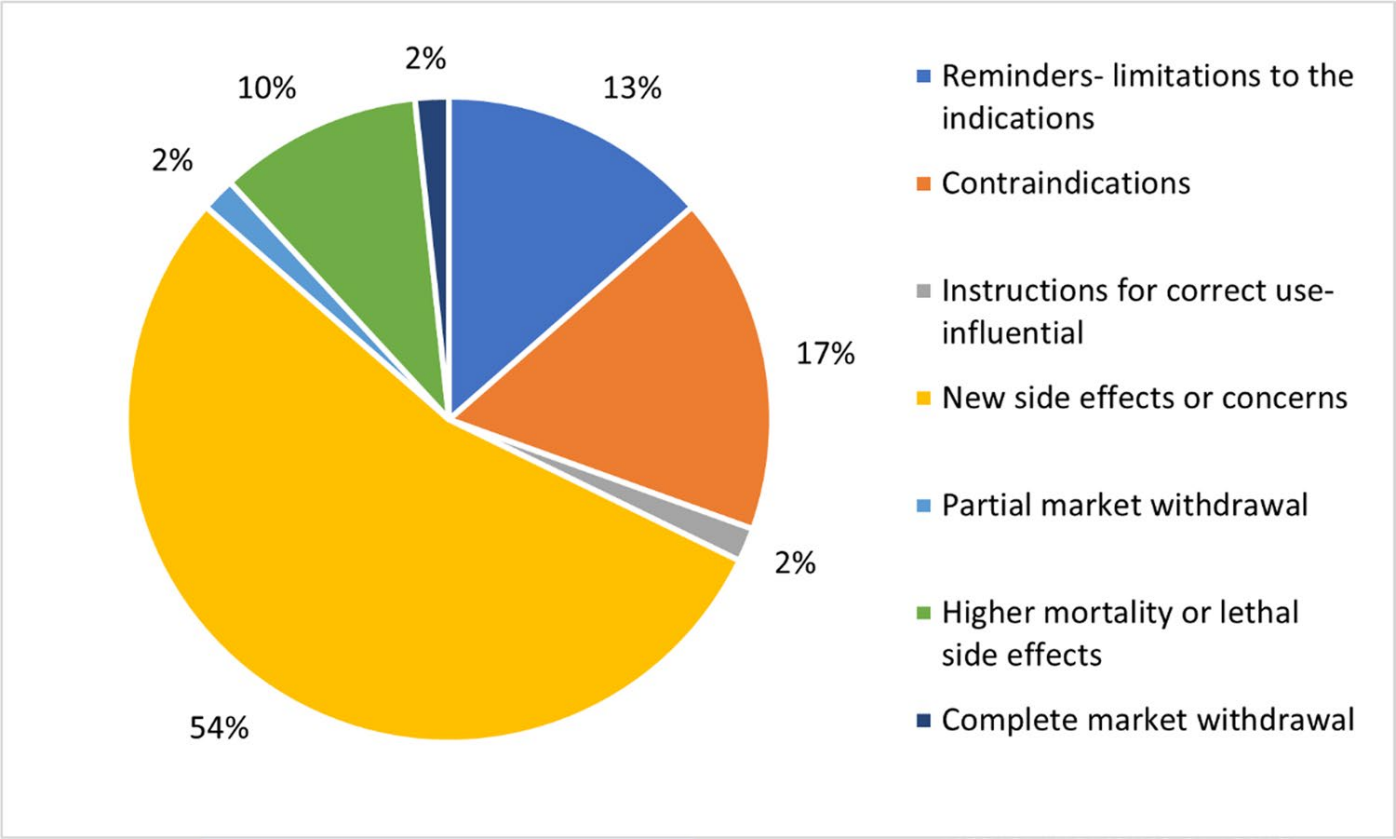
Content of potentially influential RHBs for the successful drugs

The most common potentially influential RHBs were similar among successful drugs and the entire analyzed cohort. The predominant type of RHBs, addressing new adverse drug reactions or other similar concerns like interactions, or expanding the patient population affected by a known adverse effect (e.g., drug interactions with common medications), constituted 53.1% of the potentially influential RHBs, with a slightly higher proportion of 54.2% in the successful group. This was followed by RHBs related to new contraindications (14.8%, compared to 16.9% in the successful group) and increased mortality (12.3%, with 10% in the successful group; reminders of the utility spectrum or limitations of drug use were the third most common in the successful group). The least common potentially influential RHBs were those concerning a partial market withdrawal (where only the sirup form was concerned due to quality issues), with only one case observed overall. This was followed by complete market withdrawal (3.7% in the whole contingent) (Figs. 12 and 13).

Special emphasis should be placed on findings indicating severe adverse drug reactions and/or their consequences (higher mortality, partial or complete market withdrawal), which should generally have a direct negative impact on sales. This relationship was previously demonstrated in the Netherlands for the period 2000–2008, where both short- and long-term significant reductions in drug use were observed following safety-related regulatory actions as a result of RHBs or their equivalents (Sigrid et al. 2012)

It is also worth mentioning drugs accumulating potentially influential RHBs over the years, such as lenalidomide (7RHBs, 6 of them about new adverse drug reactions), thalidomide (5 RHBs, 4 of them about new adverse drug reactions), or fingolimod (9 RHBs, some of them about potentially lethal adverse drug reactions or new contraindications). That accumulation of RHBs over time was correlated with a slowed or halted progression is noticeable in the top 3000 list as well as in the number of sales (in the case of fingolimod and lenalidomide) or with a decline (in the case of thalidomide).

Regarding the timing of potentially influential RHB publications, we observed no clear trend (such as a steady increase or decrease) over time. Notably, there was a peak in the number of RHBs in the second year after a drug’s market entry, following a minimum during the year of approval, when excluding one RHB issued before approval (for Epoetin zeta, with the RHB targeting many drugs of its class). This observation was consistent across both the entire analyzed cohort and the successful drug group (Figs. S2 and S3, in the supplement).

### Defined daily doses-TOPs and FLOPs

The following tables were obtained after thorough filtering of the data contingent provided by the WidO and identification of the 10 most successful (top 10, Table 2) as well as the 10 least successful (flop 10, Table 3) drugs, as explained in the materials and methods section. In these tables, green indicates the best performance, and yellow and orange, respectively, indicate moderately good and poor performance. Properties of the different drugs were obtained from expert opinions expressed in the corresponding AVRs, with additional information from online databases arznei-telegramm (https://www.arznei-telegramm.de/01index.php3, accessed October 18, 2024, used mainly for indications) and pharmazeutische Zeitung (https://www.pharmazeutische-zeitung.de/, pharmaceutical magazine, accessed October 18, 2024).

**Table 2:**
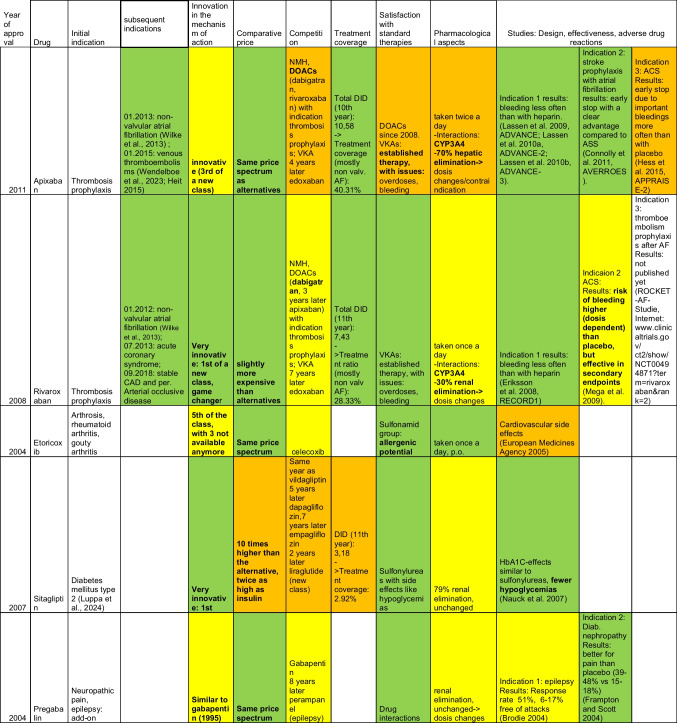

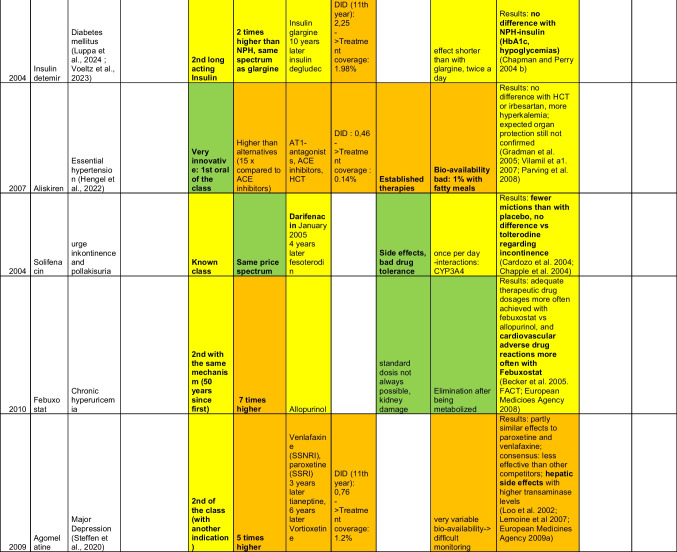
Overview of the top 10 drugs, based on DDD numbers

**Table 3:**
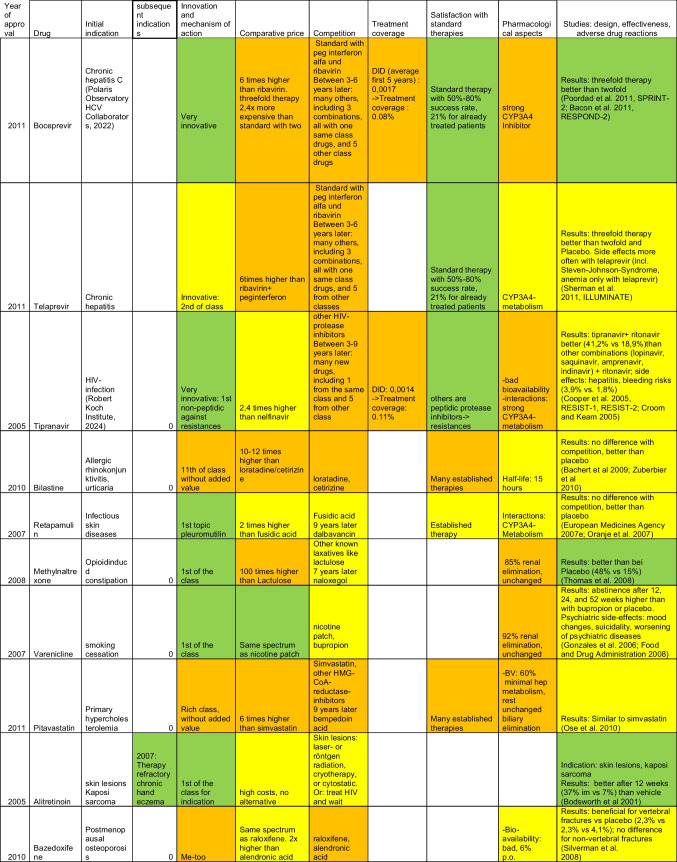
Overview of the flop 10 drugs, based on DDD numbers

### Year of release

Among the top 10 drugs, 4 were released in 2004, 2 in 2007, and 1 each in 2008, 2009, 2010, and 2011.

For the flop 10 drugs, 3 were released in 2011, while 2 were released each in 2005, 2007, and 2010 and 1 in 2008.

### Indications

Among the top 10 drugs, 2 obtained approval for additional indications beyond their initial ones, both from the direct oral anticoagulant (DOAC) class. Apixaban, initially approved in 2011 for thrombosis prophylaxis following hip and knee replacements, received further indications for non-valvular atrial fibrillation in 2013 and venous thromboembolism in 2015. Similarly, rivaroxaban, initially approved in 2008 for thrombosis prophylaxis, was later approved for non-valvular atrial fibrillation in 2012, acute coronary syndrome in 2013, and stable coronary artery disease as well as peripheral arterial vascular disease in 2018.

Additionally, pregabalin (for neuropathic pain and as an adjunct for focal epileptic seizures), etoricoxib (for osteoarthritis, rheumatoid arthritis, and gouty arthritis), and solifenacin (for pollakiuria and urge incontinence) are notable multi-purpose drugs, all of which were approved in 2004. Unlike the DOACs, these drugs were introduced with their full range of indications and did not receive additional approvals thereafter.

Among the flop 10 drugs, only one has received approval for a second indication: alitretinoin, initially approved in 2005 for skin lesions related to Kaposi sarcoma, and later obtained approval for therapy-resistant chronic hand eczema. Additionally, it is noteworthy that two drugs for hepatitis C treatment were among the “flops,” as well as two drugs for HIV-related conditions: alitretinoin (originally for Kaposi sarcoma) and tipranavir (for HIV treatment).

From an indication standpoint, we note the advantage of targeting high-prevalence chronic diseases, which we expected. To further assess the potential relation between the prevalence of underlying indications and specific drug sales, further research is essential. Such studies would not only deepen our understanding of this relationship but also provide valuable insights from a pharmacoeconomic perspective, particularly in analyzing the market share of new drugs. Additionally, they could establish a new benchmark for evaluating drug success in the future.

It is also interesting to notice the advantage that multi-purpose drugs can offer (5 out of 10 in the top 10 vs 1 out of 10 in the flop 10), especially when targeting many high-prevalence diseases. Repositioning of drugs in those cases may offer many advantages, such as cost-effectiveness and the possibility of bypassing the lengthy development process typically required for new drugs, moving directly to preclinical testing and clinical trials, which reduces both risk and costs (Guangxu et al. 2014).

### Competition

Among the top 10 drugs, 6 faced moderate competition, generally contending with one other established drug offering similar or potentially similar advantages (e.g., oral anticoagulants). The remaining 4 encountered relatively strong competition, with two or more alternative drugs providing comparable benefits for the same indications. The case of sitagliptin is particularly noteworthy, as it faced competition from a drug approved in the same year (vildagliptin), and from the first GLP-1 inhibitor, which was approved two years after sitagliptin. Despite this, no clear pattern of slowed growth or decline in sales was observed in the subsequent years based on the data obtained from WidO (Tab S1 in the supplement).

Six drugs had promising prospects due to the known therapeutic limitations of existing options, even in the presence of other drugs with the same mechanism of action (e.g., sitagliptin and rivaroxaban). In contrast, 2 drugs had less favorable prospects: apixaban, despite being in a new class, faced strong competition from established therapeutic options with no significant issues addressed by the drug; and aliskiren, which had well-established indications and effective therapeutic alternatives without major unresolved issues. The remaining 2 drugs did not have their prospects assessed in the AVR.

In terms of treatment coverage, apixaban had the highest percentage at 40.31%, followed by rivaroxaban at 28.33%, primarily for patients with non-valvular atrial fibrillation, with other indications for both drugs, such as thrombosis prophylaxis, being typically managed in inpatient settings. However, since therapy for venous thromboembolism is often continued in outpatient settings after initiation in the hospital, these figures may represent an overestimation. Other top drugs—sitagliptin (2.92%), insulin detemir (1.98%), aliskiren (0.14%), and agomelatin (1.2%)—are underutilized in relation to the available patient population. It is important to note that insulin is not considered a first-line therapy for type 2 diabetes.

These findings highlight the growing acceptance of apixaban and rivaroxaban in outpatient settings, positioning them as potential first-line treatments, particularly for non-valvular atrial fibrillation. Meanwhile, the other top drugs are struggling to achieve similar status.

Among the flop 10, three drugs (boceprevir, telaprevir, and tipranavir) were positioned favorably due to known issues with standard treatments, such as poor outcomes (for example 50 to 80% success rate in hepatitis C treatment after 10 years and problematic drug resistance associated with HIV protease inhibitors). However, these drugs faced substantial competition soon after their approval (within 3 to 8 years), both from drugs of the same class and from other classes targeting the same indications (chronic hepatitis and HIV). This competition led to a marked decline in sales, starting in their third year on the market, following initially promising sales figures, although the decline was milder in the case of tipranavir. Four other drugs faced moderate competition, with three of them competing against established therapies and methylnaltrexone addressing therapy-resistant obstipation as an ultima ratio drug. Of those four, two (methylnaltrexone in its 7^th^ year and retapamulin in its 9^th^ year) also had to face additional competition after some years on the market, which correlated with a decline in sales for retapamulin, while methylnaltrexone showed no sign of such a decline, based on the data obtained from WidO (Tab S1 in the supplement). The remaining three drugs encountered strong competition from established, proven therapies with few therapeutic issues at the time of their approval.

In terms of treatment coverage, several drugs performed exceptionally poorly, such as boceprevir at 0.08% and tipranavir at 0.0014% of the available patient population. This highlights the minimal role these drugs play for their intended indications.

### Innovation and mechanism of action

Among the top 10 drugs, 3 were novel in terms of their mechanism of action. Four drugs were the second in their respective classes, with pregabalin being a notable example (introduced 9 years after its predecessor, gabapentin). Other examples include febuxostat, which followed nearly 50 years after the previous drug in its class (allopurinol), agomelatine (approved 1 year after the first drug in its class, although for another indication), and insulin detemir, which was introduced 4 years after its predecessor, insulin glargine. Apixaban is the third drug in its class, introduced 3 years after the first. Solifenacin is another from a relatively rich class, and etoricoxib is the fifth drug in its class but distinguishes itself by being the one with the highest specificity to its pharmacological target. Two drugs in this class were withdrawn due to cardiovascular adverse drug reactions, and a third is a prodrug of one of the withdrawn drugs, leaving only celecoxib, which was approved 4 years earlier.

Six of the bottom 10 drugs could be considered innovative with respect to their mechanism of action. This includes tipranavir, which, although part of the HIV protease inhibitor class, is notable for being the first non-peptidic inhibitor, potentially mitigating drug resistance issues seen with other drugs in this class. The two drugs approved for hepatitis C treatment were released in the same year as the first drugs in their class. The remaining drugs in the bottom 10 were either “me-too” drugs or belonged to established classes without offering significant additional value.

This shows that innovation based purely on the mechanism of action should not be seen as a guarantee for creating “blockbuster” drugs. It underscores the necessity to evaluate drugs beyond that only metric and to consider other factors that can influence the sales of a newly approved drug.

Furthermore, pharmaceutical companies have long been criticized for not focusing on useful therapeutic areas with a potential for higher sales, despite the need for better therapies. In fact, only one out of 20 drugs introduced in 2012 reached the highest rating for demonstrating innovative therapeutic advances (Stafford 2015). An equivalent observation would also hold for drugs developed earlier, between 1995 and 2003 (Motola et al. 2006). This calls for a reflection as to how to improve innovation-focused R&D productivity, for instance with better target validation or reduction of attrition costs in development phases (Steven et al. 2010).

### Costs

Among the top 10 drugs, 4 had significantly high costs, exceeding existing therapies by more than five times. Four drugs were priced in the same segment as existing therapies, while 2 drugs were moderately more expensive, costing 2 to 5 times more than their counterparts.

Interestingly enough, 3 out of the 4 drugs with significantly high costs (aliskiren, febuxostat, and agomelatine) were facing well-established therapies. Two of them had little additional value in the sense of mechanism of action, and aliskiren was released for essential hypertonia, competing with alternatives that had proven to be very satisfactory while costing 15 times less in the case of ACE inhibitors.

The bottom 10 drugs were relatively costly. Only one was priced similarly to the standard available therapies, while 5 were 2 to 5 times more expensive, and an additional 4 drugs were over 5 times more expensive than the standard treatments.

Based on these data, the real game changers were priced reasonably, while other drugs with marginal added value were severely overpriced. This observation is even more pronounced when examining the drugs that failed to perform well. It was observed that overpricing often applies to new drugs with limited innovation in their mechanism of action and indications that are already well-served by established therapies, rendering this phenomenon difficult to justify (Haserück et al. 2022). This trend of inflated prices without correlation with clinical benefit makes action for price regulation necessary, which was the aim of the AMNOG (German Pharmaceutical Market Reorganization Act) introduced in 2011 (Dingermann 2013).

Ten years after the implementation of the law, potential savings are significant, especially for drugs without added benefit, amounting to around € 523.5 million (Kleining et al. 2023). Nonetheless, excessive pricing remains an important issue that needs to be tackled while maintaining a balance between innovation incentives and affordability (Akker and Sauter 2022).

### Pharmacological aspects

Aliskiren’s low bioavailability, which drops to 1% when taken with fatty meals, presents a potential drawback that has been discussed since the drug’s approval. Although the drug has achieved a placement among the top 10, it is important to mention its progressive fall after an excellent start in the first 5 years (232 th in the 5^th^ year) on the market, losing on average 137 places per year for the next 6 years to reach the 1059^th^ place in the 11^th^ year.

Two other top drugs had major pharmacological concerns: apixaban, due to interactions with CYP3A4 as well as its hepatic elimination and the necessity for dose changes or replacement with another drug in case of poor organ function, and agomelatine, with its variable bioavailability, making the drug response difficult to assess.

Five drugs among the flop 10 also had significant pharmacological concerns: 2 of these had strong interactions with CYP3A4, and 3 were primarily eliminated through renal and/or hepatic pathways, with minimal or no metabolism beforehand. Two drugs had moderate CYP3A4 metabolism, while 1 drug, bazedoxifene, was notable for its poor bioavailability coupled with a relatively long half-life of 28 h.

### Studies at the time of drug approval

The studies concerning the tops referenced in the AVRs investigated a total of 15 indications. Among these, positive results were reported for 7 indications: 5 showed better efficacy compared to alternative therapies, and 2 were more effective than placebo. Neutral results were observed for 4 indications, where the drug was neither superior nor objectively inferior to existing treatments. Negative outcomes were noted in 2 cases, where the drugs either had reduced efficacy or significant adverse effects. Additionally, 1 study was interrupted early (apixaban), and 1 study remained inconclusive by the time of publication of the corresponding AVR.

As for the flops, three drugs demonstrated very positive results in studies, particularly in regards to patient outcomes. In contrast, the remaining seven either showed no improvement over placebo or were associated with significant adverse drug reactions, such as hepatitis, bleeding (e.g., Tipranavir), mood changes, and suicidality (e.g., varenicline).

Most of the flops and some of the tops were not involved in clinical studies assessing their added value compared to standard care. This phenomenon could also be observed in other new drugs, particularly those introduced in 2009 and 2010 where only 28% of new drugs were assessed against a standard alternative (Glaeske 2012). This was later resolved with the introduction of early benefit assessments, which allowed more transparency and focused on added value. A significant majority of drugs from 2011 to 2017 (53%) were then found to have no added benefit compared to standard care (Wieseler et al. 2019). Given this concerning trend, in which numerous drugs are approved with limited evidence of their effectiveness, regulators should require more robust evidence from larger, longer-term trials before approving new drugs.

## Limitations

The analyses in this study are based exclusively on publicly available information, particularly on the AVRs as well as data from the WidO. Therefore, only apparent discrepancies can be shown. In addition, the prescription, sales, and DDD data refer only to drugs prescribed by physicians for outpatient use and dispensed via public pharmacies at the expense of the statutory health insurance system. Drugs prescribed via private health insurance and drugs prescribed in hospitals or other inpatient settings are therefore not included in this analysis. Furthermore, the analyzed drugs may have encountered different market conditions, having been approved over 8 years. These could for example encompass the regulatory environment, public and physician perception, or other political influences not discussed in our analysis. Finally, more studies are needed to validate our results on the effect of RHBs, and to analyze market shares, possibly establishing a new tool to measure drug success in the future.

## Conclusion and take-home messages

In conclusion, our analysis of 190 drugs over an 11-year period revealed that a significant portion (49%) met our success criterion. Notably, most of these successful drugs achieved this early on, with 66% meeting the criterion by the end of their second year on the market and continuing to perform well over time. Out of the 190 drugs we analyzed, 30% received RHBs, with most of them (84%) receiving at least one potentially influential RHB. Interestingly, successful drugs were more frequently subject to these than non-successful drugs, possibly because successful drugs were more often utilized than non-successful ones, allowing physicians to detect and signal issues that were uncovered until then. Most of the potentially influential RHBs were related to adverse drug reactions, indications, or contraindications, with market withdrawals being absolute exceptions.

Many factors can influence market success, making a prognosis for success in the case of a specific drug challenging. Such predictions should therefore always be based on a multifactorial approach that includes the indication(s), innovation, the competitive landscape, the comparative costs, pharmacological aspects as well as studies regarding the efficacy and the adverse drug reactions of the drug. Targeting high-prevalence chronic diseases that are underserved by currently available therapies with innovative medications can be a recipe for success. Conversely, targeting well-served indications without known issues, or getting approval for a costly medication without significant added value, often results in disappointing sales. An intriguing observation from our study was the performance of some drugs that initially succeeded despite offering limited added value, only to experience rapid declines in the long term. That observation can be interpreted as an adaptability of the market and its actors. This should encourage pharmaceutical companies to focus primarily on objective factors like added value, proof of efficacy in studies against standard therapies, costs, and practicality.

## Supplementary Information

Below is the link to the electronic supplementary material.

Supplementary file1 (DOCX 207 KB)

## Data Availability

All source data for this study are available upon reasonable request from the authors.

## Abbreviations

AMNOG: Arzneimittelmarktneuordnungsgesetz (German Pharmaceutical Market Reorganization Act)
AkdÄ: Arzneimittelkommission der deutschen Ärzteschaft (Drug Commission of the German Medical Association)
AVR: Arzneiverordnungsreport (Drug prescription report)
BfarM: Bundesinstitut für Arzneimittel und Medizinprodukte (Federal Institute for Drugs and Medical Devices)
DID: Defined daily doses prescriptions per 1000 inhabitants per day
EMA: European Medicines Agency
Mabs: Monoclonal antibodies
RHB(s): Rote-Hand-Briefe (Direct Healthcare Professional Communication, DHPC)
Tinibs: Tyrosinkinase inhibitors
WidO: Wissenschaftliches Institut der Ortskrankenkassen (AOK) (Scientific Institute of the General Local Health Insurance Fund, AOK)
GBA: Gemeinsamer Bundesausschuss (Federal Joint Committee)
