# Correction to: New drugs and their performance 10 years after approval: a systematic analysis

**DOI:** 10.1007/s00210-026-05372-z

**Published:** 2026-05-08

**Authors:** Bores Manfouo, Roland Seifert

**Affiliations:** Institute of Pharmacology, Hannover Medical School, 30625 Hannover, Germany

**Correction to: Naunyn-Schmiedeberg's Archives of Pharmacology (2025) 398:15515–15534**

10.1007/s00210-025-04178-9

In the original publication: Manfouo, B., & Seifert, R. (2025). New drugs and their performance 10 years after approval: a systematic analysis. *Naunyn–Schmiedeberg's archives of pharmacology*, *398*(11), 15,515–15,534. 10.1007/s00210-025-04178-9, several DOI links in the reference list were incorrect. This error was likely due to the simultaneous use of many reference management softwares. We sincerely apologize for this and provide an updated reference list in this erratum.

To ensure the accuracy of the revised references, each cited article was carefully verified, and all DOI links were corrected and standardized in format. Additionally, the citation style was thoroughly reviewed, and minor inconsistencies—such as inaccuracies in the ordering of authors’ names—were rectified.

- **References**
  Akker I, Sauter W (2022) Excessive pricing of pharmaceuticals in EU law: Balancing competition, innovation and regulation. In Parcu PL, Monti G, Botta M (eds), The Interaction of Competition Law and Sector Regulation : Emerging Trends at the National and EU Level (pp. 233–258). Edward Elgar Publishing Ltd.. 10.4337/9781800888708.00019
- Bachert C, Kuna P, Sanquer F, Ivan P, Dimitrov V, Gorina MM, van de Heyning P, Loureiro A, Bilastine International Working Group (2009) Comparison of the efficacy and safety of bilastine 20 mg vs desloratadine 5 mg in seasonal allergic rhinitis patients. Allergy 64(1):158–165. 10.1111/j.1398-9995.2008.01813.x
- Bacon BR, Gordon SC, Lawitz E, Marcellin P, Vierling JM, Zeuzem S, Poordad F, Goodman ZD, Sings HL, Boparai N, Burroughs M, Brass CA, Albrecht JK, Esteban R; HCV RESPOND-2 Investigators. (2011) Boceprevir for previously treated chronic HCV genotype 1 infection. New England J Med 364(13):1207–1217. 10.1056/NEJMoa1009482
- Becker MA, Schumacher HR Jr, Wortmann RL, MacDonald PA, Eustace D, Palo WA, Streit J, Joseph-Ridge N (2005) Febuxostat compared with allopurinol in patients with hyperuricemia and gout. N Engl J Med 353(23):2450–2461. 10.1056/NEJMoa050373
- Bodsworth NJ, Bloch M, Bower M, Donnell D, Yocum R, International Panretin Gel KS Study Group (2001) Phase III vehicle-controlled, multi-centered study of topical alitretinoin gel 0.1% in cutaneous AIDS-related Kaposi's sarcoma. Am J Clin Dermatol 2(2):77–87. 10.2165/00128071-200102020-00004
- Brodie MJ (2004) Pregabalin as adjunctive therapy for partial seizures. Epilepsia 45(Suppl 6):19–27. 10.1111/j.0013-9580.2004.455004.x
- Cardozo L, Lisec M, Millard R, van Vierssen Trip O, Kuzmin I, Drogendijk TE, Huang M, Ridder AM (2004) Randomized, double-blind placebo controlled trial of the once daily antimuscarinic agent solifenacin succinate in patients with overactive bladder. J Urol 172(5 Pt 1):1919–1924. 10.1097/01.ju.0000140729.07840.16
- Chapman TM, Perry CM (2004) Insulin detemir: a review of its use in the management of type 1 and 2 diabetes mellitus. Drugs 64(22):2577–2595. 10.2165/00003495-200464220-00008
- Chapple CR, Rechberger T, Al-Shukri S, Meffan P, Everaert K, Huang M, Ridder A, YM-905 Study Group (2004) Randomized, double-blind placebo- and tolterodine-controlled trial of the once-daily antimuscarinic agent solifenacin in patients with symptomatic overactive bladder. BJU Intl 93(3):303–310. 10.1111/j.1464-410x.2004.04606.x
- Cooper D et al. (2005) 24-week RESIST study analyses: The efficacy of tipranavir/ritonavir (TPV/r) is superior to lopinavir/ritonavir (LPV/r), and the TPV/r treatment response is enhanced by inclusion of genotypically active antiretrovirals in the optimized background regimen (OBR). *12th Conference on Retroviruses and Opportunistic Infections*, Boston, February 22–25, 2005. Available at https://www.natap.org/2005/CROI/croi_6.htm
- Croom KF, Keam SJ (2005) Tipranavir: a ritonavir-boosted protease inhibitor. Drugs 65(12):1669–1679. 10.2165/00003495-200565120-00005
- Connolly SJ, Eikelboom J, Joyner C, Diener HC, Hart R, Golitsyn S, Flaker G, Avezum A, Hohnloser SH, Diaz R, Talajic M, Zhu J, Pais P, Budaj A, Parkhomenko A, Jansky P, Commerford P, Tan RS, Sim KH, Lewis BS, Van Mieghem W, Lip GY, Kim JH, Lanas-Zanetti F, Gonzalez-Hermosillo A, Dans AL, Munawar M, O’Donnell M, Lawrence J, Lewis G, Afzal R, Yusuf S; AVERROES Steering Committee and Investigators (2011) Apixaban in patients with atrial fibrillation. The New England J Med 364(9):806–817. 10.1056/NEJMoa1007432
- Dingermann T (2013) Das Arzneimittelmarktneuordnungsgesetz (AMNOG) und seine Folgen [The AMNOG and its consequences]. Internist 54(6):769–774. 10.1007/s00108-013-3247-2
- Eriksson BI, Borris LC, Friedman RJ, Haas S, Huisman MV, Kakkar AK, Bandel TJ, Beckmann H, Muehlhofer E, Misselwitz F, Geerts W, RECORD1 Study Group (2008) Rivaroxaban versus enoxaparin for thromboprophylaxis after hip arthroplasty. The New England J Med 358(26):2765–2775. 10.1056/NEJMoa0800374
- European Medicines Agency (2005) European Medicines Agency announces regulatory action on COX-2 inhibitors. *Public Statement EMEA/62838/2005*. Retrieved from https://www.emea.europa.eu
- European Medicines Agency (2007) Altargo. *European Medicines Agency*. Retrieved from https://www.emea.europa.eu/humandocs/PDFs/EPAR/altargo/H-757-en6.pdf
- European Medicines Agency (2008) CHMP Assessment Report for Adenuric. *European Medicines Agency*. Retrieved from https://www.ema.europa.eu/docs/en_GB/document_library/EPAR_Public_assessment_report_human/000777/WC500021815.pdf
- European Medicines Agency (2009) Assessment report for Valdoxane. *European Medicines Agency*. Retrieved from https://www.emea.europa.eu/humandocs/PDFs/EPAR/valdoxan/H-915-en6.pdf
- Food and Drug Administration (2008) Public health advisory: Important information on Chantix (varenicline). *FDA*. Retrieved from https://www.fda.gov/cder/drug/advisory/varenicline.htm
- Frampton JE, Scott LJ (2004) Pregabalin: in the treatment of painful diabetic peripheral neuropathy. Drugs 64(24):2813–2821. 10.2165/00003495-200464240-00006
- Garattini L, Finazzi B, Mannucci PM (2022) Pharmaceutical pricing in Europe: time to take the right direction. Intern Emerg Med 17(4):945–948. 10.1007/s11739-022-02960-8
- Germany Trade & Invest (2024) *Pharmaceutical industry*. Retrieved October 18, 2024, from https://www.gtai.de/en/invest/industries/healthcare-market-germany/pharmaceutical-industry#toc-anchor--1
- Glaeske G (2012) The dilemma between efficacy as defined by regulatory bodies and effectiveness in clinical practice. Deutsches Arzteblatt Intl 109(7):115–116. 10.3238/arztebl.2012.0115
- Gonzales D, Rennard SI, Nides M, Oncken C, Azoulay S, Billing CB, Watsky EJ, Gong J, Williams KE, Reeves KR; Varenicline Phase 3 Study Group (2006) Varenicline, an alpha4beta2 nicotinic acetylcholine receptor partial agonist, vs sustained-release bupropion and placebo for smoking cessation: A randomized controlled trial. JAMA 296(1):47–55. 10.1001/jama.296.1.47
- Gradman AH, Schmieder RE, Lins RL, Nussberger J, Chiang Y, Bedigian MP (2005) Aliskiren, a novel orally effective renin inhibitor, provides dose-dependent antihypertensive efficacy and placebo-like tolerability in hypertensive patients. Circulation 111(8):1012–1018. 10.1161/01.CIR.0000156466.02908.ED
- Jin G, Wong ST (2014) Toward better drug repositioning: prioritizing and integrating existing methods into efficient pipelines. Drug Discovery Today 19(5):637–644. 10.1016/j.drudis.2013.11.005
- Haserück A, Lau T, Osterloh F (2022) Preise steigen schneller als der Nutzen. Deutsches Ärzteblatt 119, 48. https://www.aerzteblatt.de/pdf/119/48/a2128.pdf. Accessed 23rd October 2024
- Heit JA (2015) Epidemiology of venous thromboembolism. Nat Rev Cardiol 12(8):464–474. 10.1038/nrcardio.2015.83
- Hengel FE, Sommer C, Wenzel U (2022) Arterial Hypertension. Dtsch Med Wochenschr 147(07):414–428. 10.1055/a-1577-8663
- Hess CN, James S, Lopes RD, Wojdyla DM, Neely ML, Liaw D, Hagstrom E, Bhatt DL, Husted S, Goodman SG, Lewis BS, Verheugt FWA, De Caterina R, Ogawa H, Wallentin L, Alexander JH (2015) Apixaban plus mono versus dual antiplatelet therapy in acute coronary syndromes: insights from the APPRAISE-2 trial. J Am Coll Cardiol 66(7):777–787. 10.1016/j.jacc.2015.06.027
- Hofbauer-Milan V, Fetzer S, Hagist C (2023) How to predict drug expenditure: a Markov model approach with risk classes. Pharmacoeconomics 41(5):561–572. 10.1007/s40273-023-01240-3
- Kaier K, Fetzer S (2015) Das Arzneimittelmarktneuordnungsgesetz (AMNOG) aus ökonomischer Sicht [The German Pharmaceutical Market Reorganization Act (AMNOG) from an economic point of view]. Bundesgesundheitsblatt Gesundheitsforsch Gesundheitsschutz 58(3):291–297. 10.1007/s00103-014-2116-z
- Kleining K, Laufenberg J, Thrun P, Ehlert D, Wasem J, Bartol A (2024) Ten years of German benefit assessment: price analysis for drugs with unproven additional benefit. Health Econ Policy Law 19(2):216–233. 10.1017/S1744133123000117
- Lassen MR, Raskob GE, Gallus A, Pineo G, Chen D, Portman RJ (2009) Apixaban or enoxaparin for thromboprophylaxis after knee replacement. N Engl J Med 361(6):594–604. 10.1056/NEJMoa0810773
- Lassen MR, Raskob GE, Gallus A, Pineo G, Chen D, Hornick P, ADVANCE-2 investigators (2010a) Apixaban versus enoxaparin for thromboprophylaxis after knee replacement (ADVANCE-2): a randomised double-blind trial. Lancet (London, England) 375(9717):807–815. 10.1016/S0140-6736(09)62125-5
- Lassen MR, Gallus A, Raskob GE, Pineo G, Chen D, Ramirez LM, ADVANCE-3 Investigators (2010b) Apixaban versus enoxaparin for thromboprophylaxis after hip replacement. N Engl J Med 363(26):2487–2498. 10.1056/NEJMoa1006885
- Lauenroth VD, Stargardt T (2017) Pharmaceutical pricing in Germany: how is value determined within the scope of AMNOG? Value in Health : J Intl Soc Pharmacoecon Outcomes Res 20(7):927–935. 10.1016/j.jval.2017.04.006
- Lemoine P, Guilleminault C, Alvarez E (2007) Improvement in subjective sleep in major depressive disorder with a novel antidepressant, agomelatine: Randomized, double-blind comparison with venlafaxine. J Clin Psychiatry 68(11):1723–1732. 10.4088/JCP.v68n1112
- Lôo H, Hale A, D’haenen H (2002) Determination of the dose of agomelatine, a melatoninergic agonist and selective 5-HT(2C) antagonist, in the treatment of major depressive disorder: a placebo-controlled dose range study. Int Clin Psychopharmacol 17(5):239–247. 10.1097/00004850-200209000-00004
- Ludwig W, Mühlbauer B, Seifert R (2022) Arzneiverordnungs-Report 2022. Springer, Berlin/Heidelberg
- Ludwig W, Mühlbauer B, Seifert R (2021) Arzneiverordnungs-Report 2021. Springer, Berlin/Heidelberg
- Luppa PB, Zeller M, Pieper M, Kaiser P, Weiss N, Vierbaum L, Freckmann G (2024) Quality assessment of glucose measurement with regard to epidemiology and clinical management of diabetes mellitus in Germany. Front Mol Biosci. 10.3389/fmolb.2024.1371426
- Paul SM, Mytelka DS, Dunwiddie CT, Persinger CC, Munos BH, Lindborg SR, Schacht AL (2010) How to improve R&d productivity: the pharmaceutical industry’s grand challenge. Nat Rev Drug Discov 9(3):203–214. 10.1038/nrd3078
- Mega JL, Braunwald E, Mohanavelu S, Burton P, Poulter R, Misselwitz F, Hricak V, Barnathan ES, Bordes P, Witkowski A, Markov V, Oppenheimer L, Gibson CM, ATLAS ACS-TIMI 46 study group (2009) Rivaroxaban versus placebo in patients with acute coronary syndromes (ATLAS ACS-TIMI 46): a randomised, double-blind, phase II trial. Lancet 374(9683):29–38. 10.1016/S0140-6736(09)60738-8
- Mintzes B, Reynolds E, Bahri P, Perry LT, Bhasale AL, Morrow RL, Dormuth CR (2022) How do safety warnings on medicines affect prescribing? Expert Opin Drug Saf 21(10):1269–1273. 10.1080/14740338.2022.2134342
- Motola D, De Ponti F, Poluzzi E, Martini N, Rossi P, Silvani MC, Vaccheri A, Montanaro N (2006) An update on the first decade of the European centralized procedure: how many innovative drugs? Br J Clin Pharmacol 62(5):610–616. 10.1111/j.1365-2125.2006.02700.x
- Møllebæk M, Kaae S, De Bruin ML, Callréus T, Jossan S, Hallgreen CE (2019) The effectiveness of direct to healthcare professional communication - a systematic review of communication factor studies. Res Social Adm Pharm 15(5):475–482. 10.1016/j.sapharm.2018.06.015
- Nauck MA, Meininger G, Sheng D, Terranella L, Stein PP, Sitagliptin Study 024 Group (2007) Efficacy and safety of the dipeptidyl peptidase-4 inhibitor, sitagliptin, compared with the sulfonylurea, glipizide, in patients with type 2 diabetes inadequately controlled on metformin alone: a randomized, double-blind, non-inferiority trial. Diabetes Obes Metab 9(2):194–205. 10.1111/j.1463-1326.2006.00704.x
- Obst CS, Seifert R (2023) Critical analysis of the prescription and evaluation of protein kinase inhibitors for oncology in Germany. Naunyn Schmiedebergs Arch Pharmacol 396(10):2529–2543. 10.1007/s00210-023-02475-9
- Obst CS, Seifert R (2025) Updated analysis of the prescription and evaluation of protein kinase inhibitors for oncology in Germany. Naunyn Schmiedebergs Arch Pharmacol 398(2):1799–1813. 10.1007/s00210-024-03377-0
- Oranje AP, Chosidow O, Sacchidanand S, Todd G, Singh K, Scangarella N, Shawar R, Twynholm M, TOC100224 Study Team (2007) Topical retapamulin ointment, 1%, versus sodium fusidate ointment, 2%, for impetigo: a randomized, observer-blinded, noninferiority study. Dermatology (Basel, Switzerland) 215(4):331–340. 10.1159/000107776
- Ose L, Budinski D, Hounslow N, Arneson V (2009) Comparison of pitavastatin with simvastatin in primary hypercholesterolaemia or combined dyslipidaemia. Curr Med Res Opin 25(11):2755–2764. 10.1185/03007990903290886
- Paffrath D, Ludwig W, Klauber J, Schwabe U (2017) Arzneiverordnungs-Report 2017: aktuelle Daten, Kosten, Trends und Kommentare. Springer, Berlin/Heidelberg
- Parving HH, Persson F, Lewis JB, Lewis EJ, Hollenberg NK, AVOID Study Investigators (2008) Aliskiren combined with losartan in type 2 diabetes and nephropathy. N Engl J Med 358(23):2433–2446. 10.1056/NEJMoa0708379
- Peinemann F, Labeit A (2019) Varying results of early benefit assessment of newly approved pharmaceutical drugs in Germany from 2011 to 2017: a study based on federal joint committee data. J Evid Based Med 12(1):9–15. 10.1111/jebm.12340
- Polaris Observatory HCV Collaborators (2022) Global change in hepatitis C virus prevalence and cascade of care between 2015 and 2020: a modelling study. Lancet Gastroenterol Hepatol 7(5):396–415. 10.1016/S2468-1253(21)00472-6
- Poordad F, McCone J Jr, Bacon BR, Bruno S, Manns MP, Sulkowski MS, Jacobson IM, Reddy KR, Goodman ZD, Boparai N, DiNubile MJ, Sniukiene V, Brass CA, Albrecht JK, Bronowicki JP, SPRINT-2 Investigators (2011) Boceprevir for untreated chronic HCV genotype 1 infection. N Engl J Med 364(13):1195–1206. 10.1056/NEJMoa1010494
- Robert Koch Institute. (2024). Epidemiological Bulletin 28/2024: Estimate of the number of new HIV infections in 2022 and 2023 and the total number of people living with HIV in Germany by the end of 2023. Retrieved from https://www.rki.de/EN/News/Publications/Epidemiologisches-Bulletin/HIV-Epid-Bull-28-2024.pdf?__blob=publicationFile&v=2
- Piening S, Reber KC, Wieringa JE, Straus SM, de Graeff PA, Haaijer-Ruskamp FM, Mol PG (2012) Impact of safety-related regulatory action on drug use in ambulatory care in the Netherlands. Clin Pharmacol Ther 91(5):838–845. 10.1038/clpt.2011.308
- Schuhmacher A, Hinder M, von Stegmann Und Stein A, Hartl D, Gassmann O (2023) Analysis of pharma R&D productivity - a new perspective needed. Drug Discovery Today 28(10):103726. 10.1016/j.drudis.2023.103726
- Schwabe U, Paffrath D (2005) Arzneiverordnungs-Report 2005: Aktuelle Daten, Kosten, Trends und Kommentare. Springer, Berlin/Heidelberg
- Schwabe U, Paffrath D (2006) Arzneiverordnungs-Report 2006: Aktuelle Daten, Kosten, Trends und Kommentare. Springer Berlin/Heidelberg
- Schwabe U, Paffrath D (2007) Arzneiverordnungs-Report 2007: Aktuelle Daten, Kosten, Trends und Kommentare. Springer Berlin/Heidelberg
- Schwabe U, Paffrath D (2008) Arzneiverordnungs-Report 2008: Aktuelle Daten, Kosten, Trends und Kommentare. Springer Berlin/Heidelberg
- Schwabe U, Paffrath D (2009) Arzneiverordnungs-Report 2009: Aktuelle Daten, Kosten, Trends und Kommentare. Springer Berlin/Heidelberg
- Schwabe U, Paffrath D (2010) Arzneiverordnungs-Report 2010: Aktuelle Daten, Kosten, Trends und Kommentare. Springer Berlin/Heidelberg
- Schwabe U, Paffrath D (2011) Arzneiverordnungs-Report 2011: Aktuelle Daten, Kosten, Trends und Kommentare. Springer Berlin/Heidelberg
- Schwabe U, Paffrath D (2012) Arzneiverordnungs-Report 2012: Aktuelle Daten, Kosten, Trends und Kommentare. Springer Berlin/Heidelberg
- Schwabe U, Paffrath D (2013) Arzneiverordnungs-Report 2013: Aktuelle Daten, Kosten, Trends und Kommentare. Springer Berlin/Heidelberg
- Schwabe U, Paffrath D (2014) Arzneiverordnungs-Report 2014: Aktuelle Daten, Kosten, Trends und Kommentare. Springer Berlin/Heidelberg
- Schwabe U, Paffrath D (2015) Arzneiverordnungs-Report 2015: Aktuelle Daten, Kosten, Trends und Kommentare. Springer Berlin/Heidelberg
- Schwabe U, Paffrath D (2016) Arzneiverordnungs-Report 2016: Aktuelle Daten, Kosten, Trends und Kommentare. Springer Berlin/Heidelberg
- Schwabe U, Paffrath D, Ludwig W, Klauber J (2018) Arzneiverordnungs-Report 2018: aktuelle Daten, Kosten, Trends und Kommentare. Springer, Berlin/Heidelberg
- Schwabe U, Paffrath D, Ludwig W, Klauber J (2019) Arzneiverordnungs-Report 2019: aktuelle Daten, Kosten, Trends und Kommentare. Springer, Berlin/Heidelberg
- Carsten S, Schwenke S (2018) Die frühe Nutzenbewertung von Arzneimitteln gemäß § 35a SGB V. 113–141. 10.1007/978-3-658-15987-0_6
- Sherman KE, Flamm SL, Afdhal NH, Nelson DR, Sulkowski MS, Everson GT, Fried MW, Adler M, Reesink HW, Martin M, Sankoh AJ, Adda N, Kauffman RS, George S, Wright CI, Poordad F, ILLUMINATE Study Team (2011) Response-guided telaprevir combination treatment for hepatitis C virus infection. The New England J Med 365(11):1014–1024. 10.1056/NEJMoa1014463
- Silverman SL, Christiansen C, Genant HK, Vukicevic S, Zanchetta JR, de Villiers TJ, Constantine GD, Chines AA (2008) Efficacy of bazedoxifene in reducing new vertebral fracture risk in postmenopausal women with osteoporosis: results from a 3-year, randomized, placebo-, and active-controlled clinical trial. J Bone Mineral Res: The Official J Am Soc Bone Mineral Res 23(12):1923–1934. 10.1359/jbmr.080710
- Stafford N. (2014). Only 3 in 20 new drugs approved in Germany in 2011 were an improvement, report says. BMJ (Clinical research ed.), 348, g2657. 10.1136/bmj.g2657
- Stafford N (2015) German drug companies are criticised for not focusing on useful therapeutic areas. BMJ (Clinical Research Ed) 351:h4938. 10.1136/bmj.h4938
- Steffen A, Thom J, Jacobi F, Holstiege J, Bätzing J (2020) Trends in prevalence of depression in Germany between 2009 and 2017 based on nationwide ambulatory claims data. J Affect Disord 271:239–247. 10.1016/j.jad.2020.03.082
- Thomas J, Karver S, Cooney GA, Chamberlain BH, Watt CK, Slatkin NE, Stambler N, Kremer AB, Israel RJ (2008) Methylnaltrexone for opioid-induced constipation in advanced illness. N Engl J Med 358(22):2332–2343. 10.1056/NEJMoa0707377
- Villamil A, Chrysant SG, Calhoun D, Schober B, Hsu H, Matrisciano-Dimichino L, Zhang J (2007) Renin inhibition with aliskiren provides additive antihypertensive efficacy when used in combination with hydrochlorothiazide. J Hypertens 25(1):217–226. 10.1097/HJH.0b013e3280103a6b
- Voeltz D, Brinks R, Tönnies T, Hoyer A (2023) Future number of people with diagnosed type 1 diabetes in Germany until 2040: an analysis based on claims data. BMJ Open Diabetes Res Care 11(2):e003156. 10.1136/bmjdrc-2022-003156
- Wendelboe AM, Raskob GE (2016) Global burden of thrombosis: Epidemiologic aspects. Circ Res 118(9):1340–1347. 10.1161/CIRCRESAHA.115.306841
- Wieseler B, McGauran N, Kaiser T (2019) New drugs: where did we go wrong and what can we do better? BMJ (Clinical Research Ed) 366:l4340. 10.1136/bmj.l4340
- Wilke T, Groth A, Mueller S, Pfannkuche M, Verheyen F, Linder R, Maywald U, Bauersachs R, Breithardt G (2013) Incidence and prevalence of atrial fibrillation: an analysis based on 8.3 million patients. Europace 15(4):486–493. 10.1093/europace/eus333
- Zuberbier T, Oanta A, Bogacka E, Medina I, Wesel F, Uhl P, Antépara I, Jáuregui I, Valiente R, Bilastine International Working Group (2010). Comparison of the efficacy and safety of bilastine 20 mg vs levocetirizine 5 mg for the treatment of chronic idiopathic urticaria: a multi-centre, double-blind, randomized, placebo-controlled study. Allergy 65(4):516–528. 10.1111/j.1398-9995.2009.02217.x

The original article has been corrected.

