# Supplementary material for: New drugs and their performance 10 years after approval: a systematic analysis
Source: Naunyn Schmiedebergs Arch Pharmacol. 2025 May 8;398(11):15515–34. doi: 10.1007/s00210-025-04178-9 (PMC12552374; doi:10.1007/s00210-025-04178-9)
Supplement: Supplementary file 1 — Supplementary file1 (DOCX 207 KB) [file 210_2025_4178_MOESM1_ESM.docx]

**Supplemental figures S1-S3**

**Bores Manfouo and Roland Seifert**

**New drugs and their performance 10 years after approval: A systematic analysis**


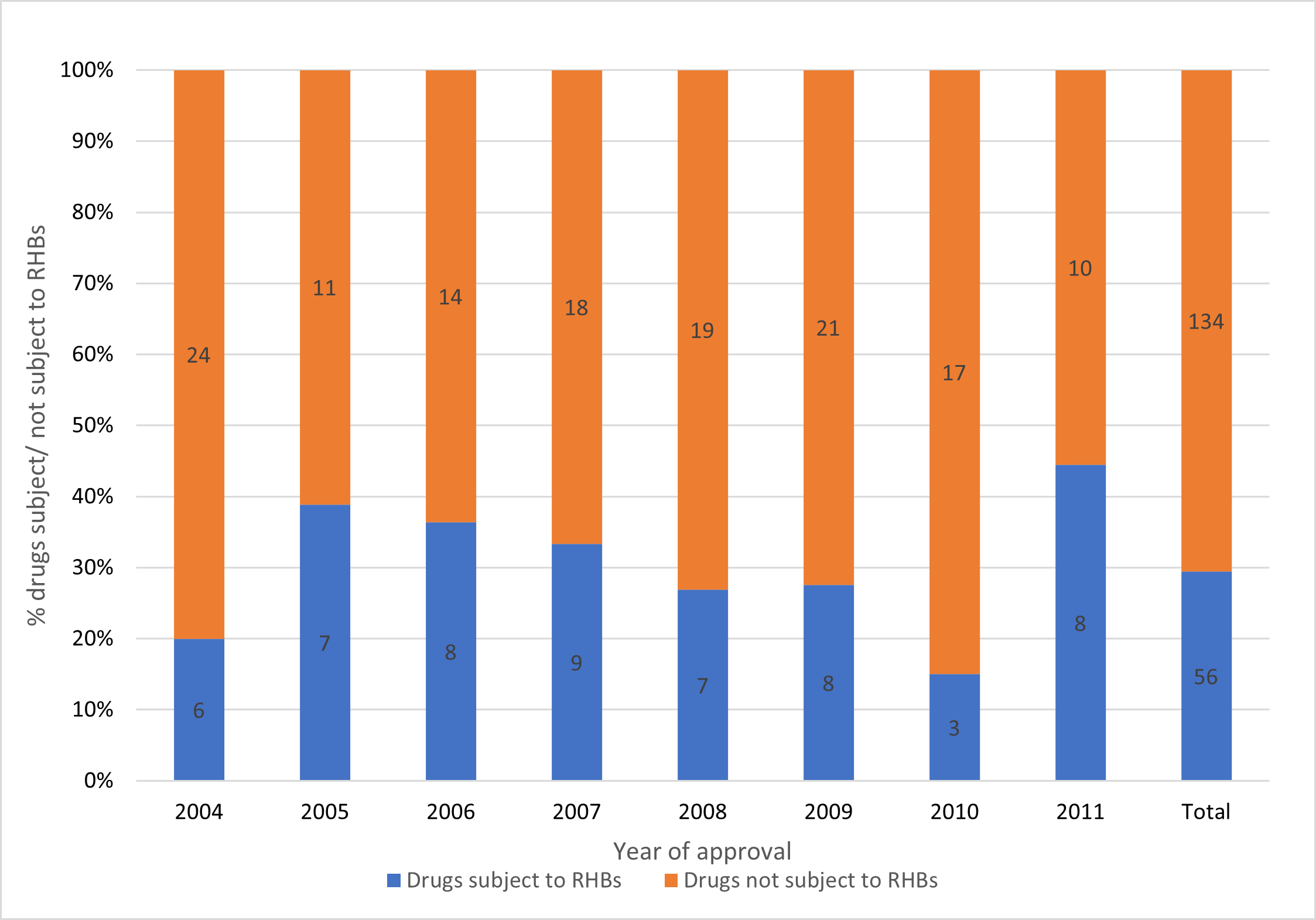


Fig. S1 Proportions of drugs subject to RHBs by year of approval (raw counts by category in bars)


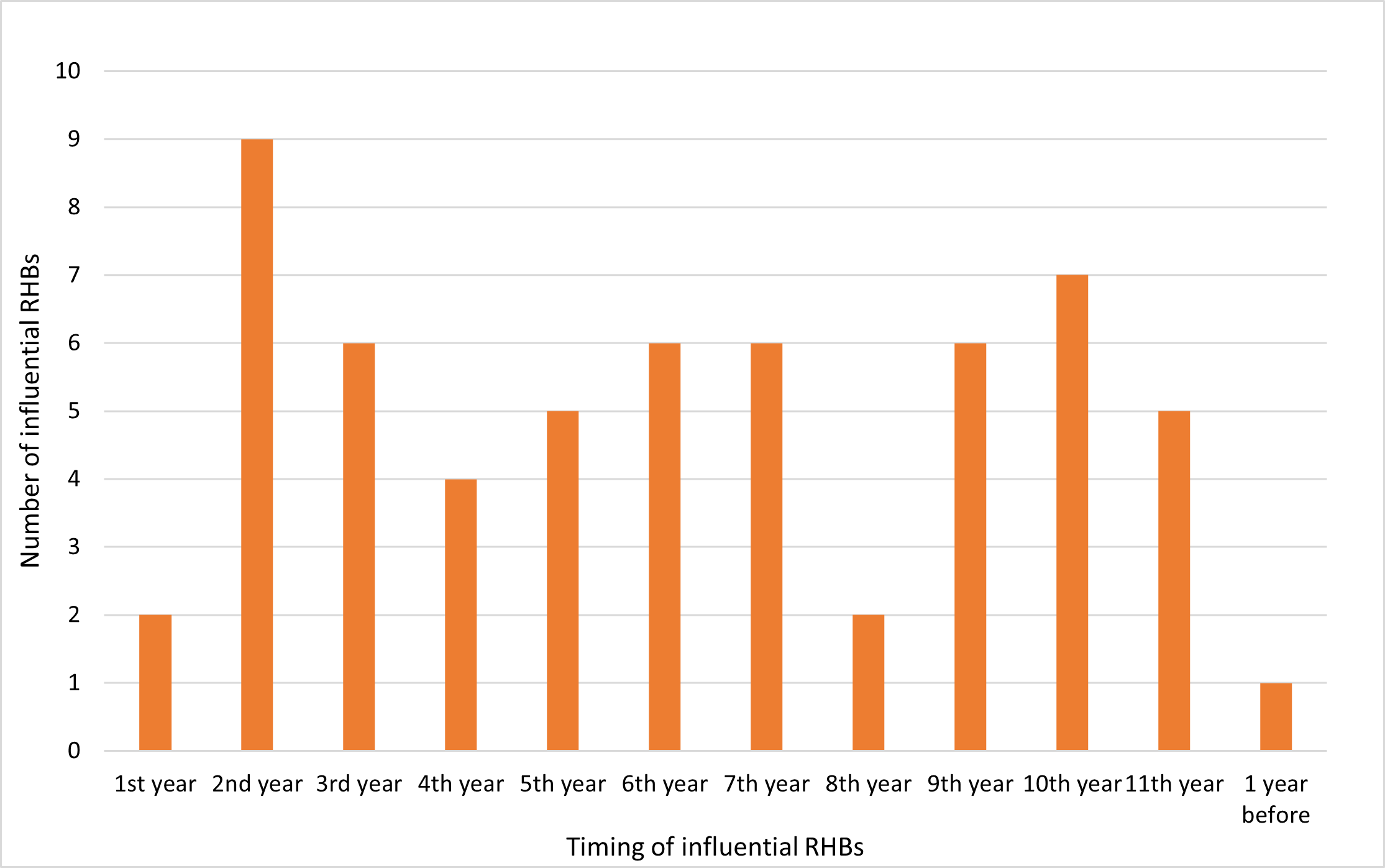


Fig. S2 Timing of influential RHBs in the successful group


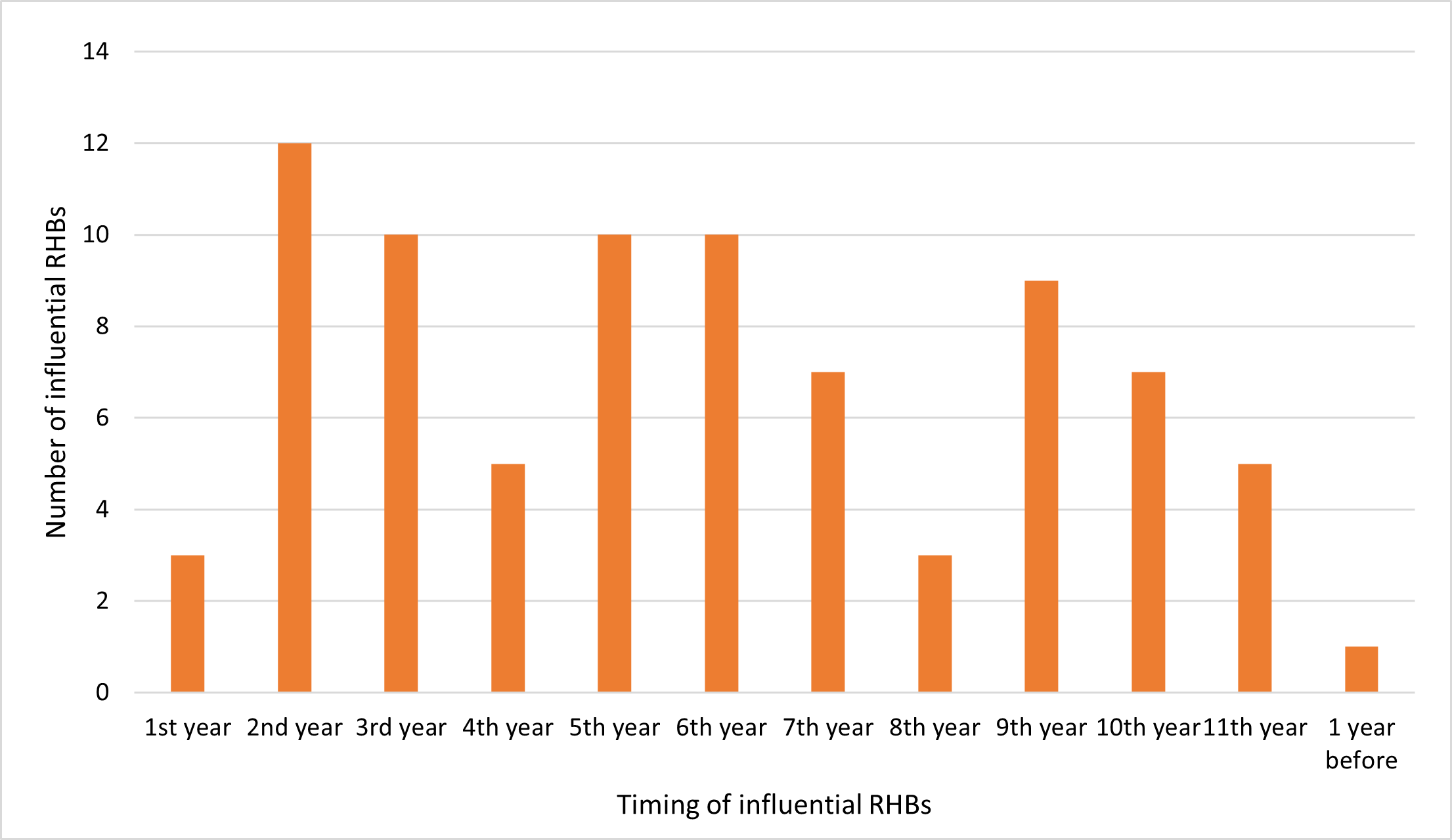


Fig. S3 Timing of influential RHBs for the whole contingent

| Approval year | Drug | 1st year | 2nd year | 3rd year | 4th year | 5th year | 6th year | 7th year | 8th year | 9th year | 10th year | 11th year | Total | Average |
| --- | --- | --- | --- | --- | --- | --- | --- | --- | --- | --- | --- | --- | --- | --- |
| 2004 | Aripiprazol | 1.040.868 | 3.733.236 | 5.019.809 | 5.950.654 | 6.826.434 | 8.014.119 | 9.695.050 | 11.298.930 | 13.113.405 | 14.595.467 | 15.901.599 | 95.189.572 | 8.653.597 |
| 2004 | Atazanavir | 614.107 | 1.091.960 | 1.169.608 | 1.221.534 | 1.319.915 | 1.593.753 | 1.681.486 | 1.839.413 | 1.796.690 | 1.740.247 | 1.546.097 | 15.614.810 | 1.419.528 |
| 2004 | Bivalirudin |  |  | 2 | 4 | 11 | 7 |  | 10 |  |  |  | 34 | 3 |
| 2004 | Bortezomib | 11.327 | 30.918 | 33.797 | 29.424 | 30.399 | 30.920 | 25.378 | 21.843 | 506.529 | 577.517 | 606.411 | 1.904.462 | 173.133 |
| 2004 | Carglumsäure | 1.498 | 5.763 | 8.779 | 5.286 | 7.135 | 6.166 | 6.620 | 6.082 | 4.716 | 3.414 | 2.464 | 57.922 | 5.266 |
| 2004 | Choleraschluckimpfstoff | 12 | 142 | 88 | 610 | 1.763 | 13.454 | 14.203 | 15.812 | 21.571 | 25.229 | 23.648 | 116.530 | 10.594 |
| 2004 | Cinacalcet | 1.352 | 679.621 | 1.297.788 | 1.694.841 | 2.064.483 | 2.396.957 | 2.609.630 | 2.786.374 | 2.819.694 | 2.884.944 | 3.054.231 | 22.289.916 | 2.026.356 |
| 2004 | Duloxetin | 604.369 | 3.483.258 | 4.204.652 | 4.433.555 | 4.225.252 | 3.866.102 | 3.560.701 | 3.330.323 | 3.152.303 | 3.006.789 | 2.918.812 | 36.786.116 | 3.344.192 |
| 2004 | Eflornithin | 1.719 | 67.870 | 110.554 | 129.269 | 118.626 | 133.483 | 128.332 | 124.783 | 117.322 | 122.761 | 133.210 | 1.187.929 | 107.994 |
| 2004 | Epinastin | 611.247 | 466.122 | 306.702 | 273.007 | 192.085 | 147.227 | 124.050 | 124.819 | 105.021 | 114.765 | 107.142 | 2.572.187 | 233.835 |
| 2004 | Eplerenon | 29.117 | 932.440 | 1.982.989 | 3.034.906 | 4.132.458 | 5.165.081 | 6.260.752 | 7.422.071 | 9.021.450 | 11.099.493 | 11.779.630 | 60.860.387 | 5.532.762 |
| 2004 | Etoricoxib | 4.250.692 | 21.158.351 | 27.886.696 | 37.238.147 | 46.582.701 | 51.725.946 | 60.357.406 | 65.131.647 | 73.319.196 | 78.560.276 | 82.678.429 | 548.889.486 | 49.899.044 |
| 2004 | Everolimus | 55.744 | 223.398 | 364.377 | 564.624 | 708.573 | 928.375 | 1.213.400 | 1.379.147 | 1.485.458 | 1.730.643 | 1.908.191 | 10.561.930 | 960.175 |
| 2004 | Fosamprenavir | 55.872 | 366.865 | 546.029 | 627.936 | 695.637 | 674.776 | 603.084 | 557.671 | 435.757 | 358.117 | 279.959 | 5.201.702 | 472.882 |
| 2004 | Fulvestrant | 164.796 | 347.177 | 407.452 | 456.665 | 519.683 | 571.510 | 860.131 | 1.284.463 | 1.408.471 | 1.416.630 | 1.537.711 | 8.974.690 | 815.881 |
| 2004 | Gadotersäure |  |  | 1 | 156 | 39 | 237 | 1.068 | 1.795 | 711 | 226 | 500 | 4.732 | 430 |
| 2004 | Insulindetemir | 2.076.752 | 12.154.090 | 17.645.733 | 24.295.637 | 31.793.847 | 37.262.750 | 41.802.393 | 47.705.313 | 55.548.360 | 61.867.531 | 64.336.005 | 396.488.409 | 36.044.401 |
| 2004 | Insulinglulisin | 213.273 | 6.681.290 | 10.081.619 | 10.889.325 | 16.317.132 | 21.302.125 | 26.372.546 | 28.653.200 | 32.210.977 | 39.252.914 | 45.629.006 | 237.603.407 | 21.600.310 |
| 2004 | Levobupivacain |  | 1.016 | 5.380 | 1.658 |  |  |  |  |  |  |  | 8.054 | 732 |
| 2004 | Manidipin | 301.249 | 3.321.174 | 2.746.528 | 1.001.643 | 775.781 | 630.087 | 529.303 | 464.635 | 396.191 | 355.382 | 318.735 | 10.840.708 | 985.519 |
| 2004 | Melagatran |  |  |  |  |  |  |  |  |  |  |  | - | - |
| 2004 | Mitotan | 1.747 | 16.961 | 24.711 | 34.609 | 35.322 | 34.271 | 36.650 | 40.185 | 44.491 | 44.909 | 51.757 | 365.615 | 33.238 |
| 2004 | Nadifloxacin | 630.932 | 946.419 | 772.090 | 748.472 | 685.738 | 678.023 | 700.516 | 652.473 | 667.224 | 673.878 | 701.123 | 7.856.888 | 714.263 |
| 2004 | Olopatadin | 1.415.709 | 1.554.121 | 1.591.272 | 1.631.574 | 1.418.785 | 1.326.333 | 1.179.730 | 1.319.599 | 1.163.824 | 1.270.918 | 1.288.228 | 15.160.093 | 1.378.190 |
| 2004 | Pemetrexed | 352 | 4.130 | 7.917 | 7.643 | 4.850 | 3.246 | 4.727 | 2.895 | 519.945 | 559.864 | 612.991 | 1.728.558 | 157.142 |
| 2004 | Pregabalin | 1.643.252 | 12.912.795 | 20.976.248 | 29.024.285 | 37.373.854 | 45.667.176 | 54.259.445 | 60.025.590 | 65.510.902 | 70.644.743 | 76.834.228 | 474.872.517 | 43.170.229 |
| 2004 | Racecadotril | 72.940 | 279.498 | 254.631 | 296.775 | 720.677 | 445.388 | 187.984 | 133.118 | 94.220 | 76.604 | 71.284 | 2.633.118 | 239.374 |
| 2004 | Solifenacin | 1.981.841 | 12.215.157 | 15.618.838 | 21.386.183 | 26.625.382 | 31.331.474 | 35.591.116 | 39.898.615 | 44.111.670 | 47.955.229 | 51.119.418 | 327.834.923 | 29.803.175 |
| 2004 | Strontiumranelat | 83.736 | 2.443.649 | 4.911.457 | 6.748.349 | 7.737.559 | 7.691.011 | 7.623.660 | 7.337.680 | 6.904.613 | 6.200.679 | 4.016.632 | 61.699.026 | 5.609.002 |
| 2004 | Ximelagatran | 49 | 97 |  |  |  |  |  |  |  |  |  | 147 | 13 |
| 2005 | Alitretinoin | 448 | 978 | 800 | 906 | 583 | 873 | 452 | 91 | 213 | 273 | 331 | 5.949 | 541 |
| 2005 | Anagrelid | 469.491 | 564.094 | 629.176 | 693.001 | 750.106 | 800.681 | 833.042 | 890.669 | 938.601 | 981.599 | 982.111 | 8.532.571 | 775.688 |
| 2005 | Argatroban | 28 | 381 | 2.019 | 5.489 | 8.795 | 9.913 | 9.956 | 12.807 | 15.892 | 19.370 | 19.863 | 104.514 | 9.501 |
| 2005 | Atomoxetin | 1.346.215 | 2.261.905 | 2.645.107 | 2.949.845 | 2.586.900 | 2.493.236 | 2.384.238 | 2.195.564 | 2.037.876 | 2.116.587 | 2.167.376 | 25.184.850 | 2.289.532 |
| 2005 | Ciclesonid | 9.671.956 | 19.270.734 | 24.998.439 | 6.236.980 | 5.420.761 | 5.530.796 | 5.261.661 | 5.184.090 | 5.269.391 | 5.289.956 | 5.306.583 | 97.441.347 | 8.858.304 |
| 2005 | Darifenacin | 4.563.435 | 7.224.879 | 9.809.704 | 12.107.245 | 13.616.360 | 15.264.484 | 15.536.581 | 14.890.738 | 14.527.335 | 13.944.741 | 12.679.841 | 134.165.343 | 12.196.849 |
| 2005 | Gadoxetsäure |  | 5 |  |  | 146 | 239 | 42 | 1 | 4 | 2 | 10 | 449 | 41 |
| 2005 | Lanreotid | 7.960 | 26.369 | 41.586 | 76.286 | 95.553 | 124.664 | 167.032 | 231.377 | 284.033 | 364.598 | 489.033 | 1.908.492 | 173.499 |
| 2005 | Loteprednol | 39.808 | 764 | 235 | 71 | 46.281 | 261.375 | 381.815 | 371.729 | 371.239 | 390.951 | 372.590 | 2.236.858 | 203.351 |
| 2005 | Natriumoxybat | 433 | 15.194 | 26.242 | 38.828 | 50.763 | 62.152 | 68.312 | 75.739 | 84.441 | 97.442 | 107.357 | 626.903 | 56.991 |
| 2005 | Nitisinon | 8.555 | 13.747 | 17.222 | 19.884 | 19.963 | 23.012 | 22.894 | 23.932 | 24.637 | 24.836 | 25.173 | 223.856 | 20.351 |
| 2005 | Palifermin | 18 | 399 | 372 | 351 | 327 | 163 | 71 | 72 | 157 | 229 | 209 | 2.367 | 215 |
| 2005 | Palonosetron | 9.937 | 33.987 | 39.510 | 44.044 | 53.428 | 70.878 | 79.490 | 93.902 | 110.730 | 130.977 | 143.242 | 810.126 | 73.648 |
| 2005 | Paricalcitol | 99.094 | 321.498 | 534.691 | 732.981 | 853.574 | 983.309 | 1.188.371 | 1.146.676 | 1.273.797 | 1.338.137 | 1.119.768 | 9.591.897 | 871.991 |
| 2005 | Posaconazol | 344 | 15.061 | 45.051 | 72.375 | 79.986 | 84.565 | 92.133 | 99.473 | 102.021 | 130.373 | 190.851 | 912.233 | 82.930 |
| 2005 | Rasagilin | 160.122 | 1.376.113 | 2.123.811 | 2.895.256 | 3.985.851 | 5.200.605 | 6.024.691 | 6.879.440 | 7.781.693 | 8.740.708 | 6.401.458 | 51.569.747 | 4.688.159 |
| 2005 | Tipranavir | 9.391 | 94.952 | 84.236 | 60.757 | 46.858 | 41.806 | 33.109 | 27.248 | 22.882 | 19.983 | 14.792 | 456.015 | 41.456 |
| 2005 | Zonisamid | 87.421 | 463.268 | 736.417 | 935.787 | 1.053.737 | 1.148.351 | 1.274.292 | 1.435.879 | 1.572.505 | 1.845.831 | 2.059.881 | 12.613.369 | 1.146.670 |
| 2006 | Alglucosidase alfa | 3.687 | 11.692 | 16.907 | 18.973 | 24.847 | 23.680 | 33.816 | 38.645 | 43.618 | 46.740 | 64.211 | 326.816 | 29.711 |
| 2006 | Carbetocin |  |  | 10 |  |  |  |  |  |  |  |  | 10 | 1 |
| 2006 | Clofarabin |  | 211 |  | 2 | 7 | 5 | 11 | 2 | 2 |  | 4 | 245 | 22 |
| 2006 | Daptomycin | 57 | 316 | 481 | 591 | 1.956 | 1.763 | 2.071 | 1.992 | 2.060 | 2.369 | 2.725 | 16.381 | 1.489 |
| 2006 | Deferasirox | 35.741 | 231.423 | 287.944 | 329.497 | 373.975 | 407.880 | 446.947 | 463.347 | 476.503 | 487.560 | 507.980 | 4.048.795 | 368.072 |
| 2006 | Dexrazoxan |  | 16 | 24 | 57 | 82 | 55 | 143 | 149 | 92 | 106 | 86 | 810 | 74 |
| 2006 | Entecavir | 50.314 | 534.674 | 1.126.691 | 1.449.488 | 1.657.094 | 1.837.599 | 1.976.572 | 2.137.762 | 2.331.838 | 2.493.215 | 2.660.139 | 18.255.386 | 1.659.581 |
| 2006 | Fomepizol |  |  |  |  |  |  |  |  |  |  |  | - | - |
| 2006 | Gadofosveset |  | 5 | 5 | 5 |  |  |  |  |  |  |  | 16 | 1 |
| 2006 | Galsulfase | 1.666 | 3.555 | 3.495 | 4.649 | 4.862 | 5.188 | 5.650 | 5.989 | 6.539 | 6.501 | 5.983 | 54.077 | 4.916 |
| 2006 | Hexaminolevulinat |  | 38 | 31 | 48 | 34 | 66 | 76 | 74 | 60 | 80 | 41 | 549 | 50 |
| 2006 | Humaner Papillom­virusImpfstoff | 139 | 239.171 | 612.773 | 373.087 | 185.774 | 225.303 | 367.331 | 324.304 | 320.126 | 381.566 | 267.654 | 3.297.229 | 299.748 |
| 2006 | Ivabradin | 409.845 | 1.636.254 | 3.499.207 | 6.319.201 | 9.689.978 | 12.784.010 | 16.331.554 | 20.332.689 | 23.703.478 | 23.003.640 | 23.796.388 | 141.506.244 | 12.864.204 |
| 2006 | Lanthancarbonat | 28.518 | 1.068.643 | 1.677.481 | 2.006.616 | 2.096.705 | 1.980.644 | 1.864.415 | 1.917.390 | 2.025.832 | 2.038.064 | 2.099.250 | 18.803.557 | 1.709.414 |
| 2006 | Parathyroidhormon,rekombiniert | 14.732 | 112.769 | 219.693 | 324.341 | 295.651 | 209.419 | 135.870 | 3.205 |  |  |  | 1.315.679 | 119.607 |
| 2006 | Rimonabant | 639.415 | 75.447 | 5.427 |  |  | 29 |  |  |  |  |  | 720.318 | 65.483 |
| 2006 | Rotavirusimpfstoff, monovalent | 57 | 159 | 1.794 | 4.829 | 47.284 | 73.630 | 127.891 | 152.922 | 65.396 | 19.048 | 7.199 | 500.209 | 45.474 |
| 2006 | Rotavirusimpfstoff, pentavalent | 11 | 103 | 1.436 | 3.562 | 64.029 | 77.037 | 110.772 | 121.944 | 44.010 | 9.542 | 2.951 | 435.399 | 39.582 |
| 2006 | Rotigotin | 511.743 | 1.939.821 | 2.792.909 | 2.764.883 | 3.470.330 | 3.900.455 | 4.462.831 | 4.862.783 | 5.318.482 | 5.730.556 | 6.057.406 | 41.812.198 | 3.801.109 |
| 2006 | Sitaxentan | 244 | 40.074 | 107.697 | 147.434 | 143.085 | 888 |  |  |  |  |  | 439.422 | 39.947 |
| 2006 | Tigecyclin | 223 | 715 | 303 | 511 | 883 | 697 | 536 | 996 | 763 | 423 | 635 | 6.685 | 608 |
| 2006 | Ziconotid | 99 | 3.625 | 12.445 | 24.144 | 36.266 | 41.692 | 51.664 | 50.106 | 47.302 | 44.576 | 38.157 | 350.075 | 31.825 |
| 2007 | Aliskiren | 1.342.365 | 18.686.192 | 42.401.764 | 58.379.806 | 73.948.775 | 47.364.980 | 29.703.702 | 23.439.890 | 18.935.926 | 15.880.293 | 13.477.484 | 343.561.176 | 31.232.834 |
| 2007 | Anidulafungin |  | 84 | 40 | 59 | 82 | 429 | 40 | 144 | 20 | 73 | 137 | 1.107 | 101 |
| 2007 | Betain | 14.875 | 25.323 | 30.397 | 32.286 | 34.947 | 34.955 | 38.926 | 44.126 | 44.733 | 49.898 | 53.858 | 404.324 | 36.757 |
| 2007 | Cilostazol | 1.410.997 | 3.038.476 | 4.495.628 | 5.554.488 | 6.090.871 | 5.995.444 | 5.269.907 | 3.961.366 | 2.027.899 | 889.803 | 549.175 | 39.284.054 | 3.571.278 |
| 2007 | Darunavir | 126.145 | 327.014 | 630.555 | 1.114.331 | 1.614.244 | 1.757.167 | 1.944.704 | 2.049.568 | 1.969.030 | 1.841.645 | 1.701.515 | 15.075.917 | 1.370.538 |
| 2007 | Epoetin delta | 800.391 | 1.061.188 | 4.274 | 6 |  |  |  |  |  |  |  | 1.865.859 | 169.624 |
| 2007 | Eptotermin alfa |  |  | 1 |  |  |  |  |  |  |  |  | 1 | 0 |
| 2007 | Exenatid | 1.658.517 | 5.753.054 | 7.660.655 | 6.313.960 | 5.929.456 | 5.633.758 | 6.317.904 | 6.602.240 | 5.495.839 | 4.373.958 | 3.385.594 | 59.124.936 | 5.374.994 |
| 2007 | Gadoversetamid |  |  |  |  |  |  |  |  |  |  |  | - | - |
| 2007 | HPV-Impfstoff |  |  |  |  |  |  |  |  |  |  |  | - | - |
| 2007 | Idursulfase | 5.510 | 7.056 | 7.299 | 6.952 | 7.847 | 8.089 | 7.161 | 8.213 | 8.435 | 9.855 | 10.539 | 86.956 | 7.905 |
| 2007 | Lenalidomid | 123.670 | 355.847 | 392.419 | 497.653 | 600.478 | 701.150 | 751.764 | 852.097 | 1.020.600 | 1.307.445 | 1.566.919 | 8.170.042 | 742.731 |
| 2007 | Maraviroc | 2.193 | 65.904 | 130.723 | 201.446 | 271.486 | 282.608 | 297.670 | 306.938 | 282.719 | 254.908 | 237.361 | 2.333.955 | 212.178 |
| 2007 | Mecasermin | 1.648 | 14.485 | 39.041 | 50.792 | 47.185 | 53.361 | 34.754 | 30.240 | 24.077 | 24.432 | 21.987 | 342.002 | 31.091 |
| 2007 | Methoxy-Polyethylenglycol-Epoetin beta |  |  |  |  |  |  |  |  |  |  |  | - | - |
| 2007 | Nelarabin |  | 17 |  |  |  | 119 | 58 | 202 | 176 | 250 | 381 | 1.202 | 109 |
| 2007 | Paliperidon | 647.151 | 2.839.098 | 3.170.824 | 70.749 | 32.530 | 68.970 | 28.895 | 26.421 | 28.297 | 26.013 | 25.949 | 6.964.897 | 633.172 |
| 2007 | Perflutren |  |  |  |  | 4 |  |  |  |  |  | 4 | 8 | 1 |
| 2007 | Retapamulin | 480 | 1.985 | 967 | 17.497 | 18.289 | 13.771 | 11.581 | 10.217 | 3.606 | 2.030 | 3.659 | 84.081 | 7.644 |
| 2007 | Rufinamid | 28.292 | 151.329 | 208.603 | 237.289 | 267.331 | 297.918 | 310.916 | 309.850 | 333.395 | 337.415 | 360.716 | 2.843.054 | 258.459 |
| 2007 | Sitagliptin | 4.885.699 | 17.177.666 | 18.438.311 | 23.803.540 | 31.192.867 | 38.462.767 | 44.295.679 | 54.754.868 | 73.084.797 | 85.543.472 | 93.023.993 | 484.663.659 | 44.060.333 |
| 2007 | Telbivudin | 37.012 | 152.256 | 188.696 | 187.149 | 178.339 | 166.173 | 135.073 | 114.172 | 95.397 | 88.900 | 74.588 | 1.417.757 | 128.887 |
| 2007 | Temsirolimus | 202 | 9.796 | 10.083 | 8.024 | 4.415 | 45.200 | 40.263 | 34.619 | 27.637 | 20.278 | 14.816 | 215.331 | 19.576 |
| 2007 | Tetrabenazin | 260.077 | 440.673 | 526.867 | 571.889 | 561.649 | 543.859 | 522.982 | 499.058 | 488.604 | 468.188 | 437.898 | 5.321.742 | 483.795 |
| 2007 | Trabectedin | 24 | 599 | 608 | 1.906 | 861 | 39.546 | 45.135 | 47.868 | 47.370 | 51.324 | 46.789 | 282.029 | 25.639 |
| 2007 | Treprostinil | 986 | 1.618 | 1.978 | 2.249 | 3.498 | 7.847 | 17.278 | 29.143 | 32.152 | 38.751 | 48.383 | 183.883 | 16.717 |
| 2007 | Vareniclin | 1.777 | 1.561 | 1.147 | 883 | 1.092 | 713 | 1.440 | 11.810 | 10.096 | 7.195 | 525 | 38.239 | 3.476 |
| 2008 | Abarelix | 44.501 | 136.272 | 121.474 | 124.225 | 114.007 | 122.450 | 47.720 |  |  |  |  | 710.649 | 64.604 |
| 2008 | Ambrisentan | 14.768 | 64.048 | 100.859 | 191.471 | 210.286 | 221.832 | 234.568 | 226.974 | 238.796 | 237.676 | 217.817 | 1.959.095 | 178.100 |
| 2008 | Aminolevulinsäure |  |  |  |  |  |  |  |  |  |  |  | - | - |
| 2008 | Antithrombin alfa |  |  |  |  |  |  |  |  |  |  |  | - | - |
| 2008 | Colesevelam | 55.325 | 87.493 | 133.062 | 178.754 | 216.005 | 235.305 | 253.386 | 257.136 | 267.587 | 277.754 | 284.002 | 2.245.808 | 204.164 |
| 2008 | Dabigatranetexilat | 12.400 | 39.089 | 100.176 | 1.994.884 | 12.463.021 | 22.930.408 | 28.693.136 | 29.801.935 | 32.104.887 | 33.734.241 | 35.018.755 | 196.892.932 | 17.899.357 |
| 2008 | Docosanol | 181 | 204 | 165 | 76 | 99 | 4 |  |  |  |  |  | 728 | 66 |
| 2008 | Doripenem |  | 52 | 13 | 13 | 3 |  |  |  |  |  |  | 81 | 7 |
| 2008 | Etravirin | 35.842 | 182.927 | 271.569 | 295.346 | 300.143 | 309.298 | 315.436 | 286.172 | 253.583 | 225.838 | 207.434 | 2.683.588 | 243.963 |
| 2008 | Fesoterodin | 456.467 | 4.465.332 | 8.114.359 | 10.675.338 | 12.019.765 | 12.682.836 | 12.972.294 | 12.966.674 | 4.607.900 | 1.103.291 | 891.955 | 80.956.211 | 7.359.656 |
| 2008 | Fluticasonfuroat | 1.039.495 | 2.197.566 | 4.225.148 | 5.867.026 | 7.714.127 | 8.862.060 | 8.058.951 | 6.545.898 | 4.988.354 | 3.447.616 | 3.356.227 | 56.302.467 | 5.118.406 |
| 2008 | Fosaprepitant |  |  |  |  |  |  |  |  |  |  |  | - | - |
| 2008 | Icatibant | 66 | 327 | 626 | 1.190 | 1.522 | 2.057 | 2.670 | 3.166 | 4.370 | 5.223 | 6.218 | 27.436 | 2.494 |
| 2008 | Lacosamid | 154.995 | 1.450.659 | 2.235.360 | 3.237.628 | 4.033.932 | 4.826.554 | 5.849.724 | 6.772.844 | 7.566.351 | 8.988.409 | 11.121.840 | 56.238.295 | 5.112.572 |
| 2008 | Laropiprant Nicotinsäure |  | 153.165 | 1.588.707 | 2.595.310 | 2.794.566 | 144.439 |  |  |  |  |  | 7.276.189 | 661.472 |
| 2008 | Melatonin | 445.156 | 1.208.523 | 1.176.075 | 1.202.776 | 1.340.310 | 1.515.669 | 1.872.549 | 2.278.472 | 2.911.622 | 3.783.201 | 4.843.128 | 22.577.481 | 2.052.498 |
| 2008 | Methylnaltrexon | 1.580 | 5.489 | 5.505 | 4.815 | 5.091 | 7.129 | 9.395 | 7.133 | 7.930 | 12.211 | 3.011 | 69.291 | 6.299 |
| 2008 | Raltegravir | 299.531 | 645.691 | 1.168.127 | 1.688.509 | 2.115.909 | 2.477.816 | 2.488.491 | 2.314.817 | 2.130.746 | 2.008.967 | 2.154.984 | 19.493.588 | 1.772.144 |
| 2008 | Rifaximin | 3.532 | 31.725 | 64.017 | 109.506 | 157.385 | 267.275 | 548.030 | 756.032 | 912.483 | 1.070.086 | 1.275.586 | 5.195.657 | 472.332 |
| 2008 | Rivaroxaban | 5.584 | 149.148 | 358.043 | 683.744 | 25.494.629 | 83.375.433 | 134.541.192 | 170.531.539 | 191.422.067 | 198.212.973 | 217.243.583 | 1.022.017.935 | 92.910.721 |
| 2008 | Rupatidin | 67.419 | 1.396.960 | 1.383.797 | 1.373.994 | 1.338.044 | 1.440.825 | 1.592.982 | 1.652.978 | 1.666.348 | 1.267.879 | 827.107 | 14.008.332 | 1.273.485 |
| 2008 | Stiripentol | 33.807 | 61.991 | 79.368 | 91.921 | 104.929 | 114.350 | 128.871 | 148.652 | 170.391 | 173.398 | 187.897 | 1.295.574 | 117.779 |
| 2008 | Sugammadex |  | 8 | 15 | 15 | 22 | 14 | 8 | 7 | 7 | 42 | 54 | 192 | 17 |
| 2008 | Tafluprost | 1.636.991 | 8.178.777 | 13.469.109 | 17.942.437 | 21.148.672 | 20.151.836 | 19.191.765 | 19.247.235 | 19.268.307 | 19.329.220 | 19.286.727 | 178.851.078 | 16.259.189 |
| 2008 | Vildagliptin | 81.748 | 675.472 | 2.145.366 | 4.034.726 | 7.665.975 | 12.692.320 | 9.432.647 | 67.606 | 488 |  | 12.678 | 36.809.026 | 3.346.275 |
|  | Zofenopril |  |  |  |  |  | 262 | 16 | 16 | 341 | 59 | 122 | 816 | 74 |
| 2009 | Agomelatin | 4.145.376 | 12.205.960 | 18.411.037 | 24.737.721 | 24.767.689 | 24.346.573 | 26.102.655 | 27.227.429 | 27.604.159 | 29.170.221 | 22.410.977 | 241.129.798 | 21.920.891 |
| 2009 | Azacitidin | 14.662 | 17.097 | 17.301 | 331.037 | 324.990 | 339.700 | 362.679 | 393.029 | 414.759 | 429.954 | 442.469 | 3.087.677 | 280.698 |
| 2009 | Dapoxetin | 176 | 182 | 104 | 111 | 72 | 217 | 127 | 88 | 151 | 131 | 89 | 1.447 | 132 |
| 2009 | Degarelix | 162.721 | 476.359 | 697.374 | 812.331 | 776.055 | 775.300 | 824.249 | 881.538 | 835.187 | 773.646 | 762.686 | 7.777.447 | 707.041 |
| 2009 | Epoetin theta |  |  |  |  |  |  |  |  |  |  |  | - | - |
| 2009 | Epoetin zeta |  |  |  |  |  |  |  |  |  |  |  | - | - |
| 2009 | Eslicarbazepin | 69.372 | 595.144 | 814.172 | 877.590 | 859.031 | 925.349 | 1.034.129 | 1.196.344 | 1.378.598 | 1.665.594 | 1.906.545 | 11.321.868 | 1.029.261 |
| 2009 | Histrelinacetat | 32.607 | 89.062 | 161.611 | 164.748 | 94.848 | 52.868 | 9.503 |  |  |  |  | 605.248 | 55.023 |
| 2009 | Inaktiviertes Virus Oberflächenantigen(H1N1), adjuvantiert |  |  |  |  |  |  |  |  |  |  |  | - | - |
| 2009 | Japanische-Enzephalitis-Virus-Impfstoff | 1.448 | 3.622 | 4.659 | 9.055 | 9.768 | 10.693 | 16.321 | 16.795 | 5.769 | 10.858 | 14.935 | 103.923 | 9.448 |
| 2009 | Liraglutid | 1.507.782 | 7.854.271 | 10.860.928 | 13.791.996 | 14.695.411 | 16.214.768 | 16.662.174 | 17.262.087 | 18.450.810 | 23.121.238 | 31.070.498 | 171.491.964 | 15.590.179 |
| 2009 | Micafungin | 15 | 62 | 71 | 135 | 165 | 297 | 436 | 273 | 1.529 | 713 | 1.117 | 4.813 | 438 |
| 2009 | PandemischerInfluenzaimpfstoff(H1N1), adjuvantiert |  |  |  |  |  |  |  |  |  |  |  | - | - |
| 2009 | PandemischerInfluenzaimpfstoffH1N1) |  |  |  |  |  |  |  |  |  |  |  | - | - |
| 2009 | PandemischerInfluenzaimpfstoff(H1N1)-Spaltvirus, inaktiviert |  |  |  |  |  |  |  |  |  |  |  | - | - |
| 2009 | PandemischerInfluenzaimpfstoff(H1N1), adjuvantiert |  |  |  |  |  |  |  |  |  |  |  | - | - |
| 2009 | Plerixafor | 72 | 104 | 81 | 74 | 122 | 104 | 135 | 140 | 154 | 146 | 259 | 1.391 | 126 |
| 2009 | Pneumokokken-Impfstoff | 771 | 1.139 | 942 | 627 | 445 | 483 | 296 | 345 | 194 | 135 | 126 | 5.502 | 500 |
| 2009 | Prasugrel | 585.117 | 3.444.614 | 6.752.658 | 10.770.828 | 13.139.241 | 13.977.407 | 14.351.994 | 13.966.256 | 12.891.343 | 11.923.378 | 8.454.198 | 110.257.032 | 10.023.367 |
| 2009 | Ranolazin | 452.698 | 1.747.340 | 2.933.521 | 5.123.126 | 7.334.498 | 9.671.966 | 11.888.788 | 13.508.422 | 14.274.934 | 15.559.231 | 17.082.280 | 99.576.803 | 9.052.437 |
| 2009 | Romiplostim | 58.435 | 170.345 | 176.820 | 235.441 | 267.689 | 313.658 | 337.572 | 393.843 | 487.246 | 544.966 | 613.983 | 3.599.998 | 327.273 |
| 2009 | Rosuvastatin | 14.194.347 | 5.059.930 | 2.797.836 | 2.119.826 | 1.625.752 | 1.599.401 | 1.504.809 | 1.532.699 | 1.655.711 | 602.684 | 268.534 | 32.961.530 | 2.996.503 |
| 2009 | Sapropterin | 9.356 | 25.729 | 38.036 | 44.163 | 47.806 | 54.314 | 58.320 | 63.313 | 71.009 | 82.552 | 92.140 | 586.738 | 53.340 |
| 2009 | Saxagliptin | 203.200 | 5.245.156 | 10.946.198 | 14.052.629 | 13.760.574 | 15.469.401 | 18.201.321 | 16.839.394 | 14.549.329 | 12.438.112 | 10.845.765 | 132.551.080 | 12.050.098 |
| 2009 | Thalidomid | 39.238 | 86.344 | 74.037 | 59.668 | 47.199 | 36.530 | 32.540 | 27.832 | 21.516 | 17.397 | 13.374 | 455.673 | 41.425 |
| 2009 | Tocofersolan | 1.620 | 7.084 | 8.839 | 11.188 | 13.173 | 17.910 | 19.800 | 20.616 | 25.187 | 26.141 | 22.326 | 173.884 | 15.808 |
| 2009 | Tolvaptan | 557 | 6.444 | 13.928 | 21.931 | 29.625 | 41.569 | 53.028 | 63.068 | 72.875 | 84.892 | 96.162 | 484.080 | 44.007 |
| 2009 | Ulipristalacetat | 528 | 8.887 | 22.843 | 27.433 | 42.902 | 51.712 | 30.003 | 16.207 | 13.891 | 11.871 | 11.868 | 238.146 | 21.650 |
|  | Vinflunin |  | 601 | 419 | 33.705 | 36.740 | 34.934 | 40.306 | 43.929 | 27.065 | 16.366 | 21.771 | 255.836 | 23.258 |
| 2010 | Amifampridin | 3.624 | 14.571 | 20.565 | 30.174 | 33.190 | 36.307 | 43.523 | 44.941 | 55.999 | 62.386 | 64.199 | 409.479 | 37.225 |
| 2010 | Asenapin |  | 38.204 | 113.865 | 132.770 | 133.624 | 124.608 | 105.296 | 97.809 | 88.092 | 82.922 | 81.222 | 998.411 | 90.765 |
| 2010 | Bazedoxifen |  |  | 85 | 84 | 88 |  |  |  |  |  |  | 257 | 23 |
| 2010 | Bilastin |  | 4.053 | 4.297 | 9.302 | 12.636 | 22.973 | 34.631 | 28.031 | 34.695 | 32.257 | 54.279 | 237.153 | 21.559 |
| 2010 | Conestat alfa |  |  | 5 | 35 | 3 | 5 | 126 | 246 | 421 | 209 | 59 | 1.110 | 101 |
| 2010 | Corifollitropin | 271 | 773 | 1.080 | 1.242 | 1.464 | 1.642 | 2.044 | 2.084 | 2.372 | 2.102 | 1.827 | 16.901 | 1.536 |
| 2010 | Dronedaron | 7.033.182 | 10.701.692 | 7.997.395 | 7.686.865 | 7.719.266 | 7.226.920 | 6.730.382 | 6.228.222 | 5.836.170 | 5.430.712 | 3.976.099 | 76.566.904 | 6.960.628 |
| 2010 | Eltrombopag | 34.108 | 96.607 | 137.364 | 191.126 | 234.526 | 297.865 | 437.740 | 563.491 | 722.109 | 863.802 | 1.039.448 | 4.618.186 | 419.835 |
| 2010 | Febuxostat | 1.694.774 | 6.975.091 | 12.616.191 | 17.717.145 | 25.663.979 | 31.792.809 | 38.577.116 | 45.243.421 | 50.869.077 | 40.676.469 | 14.306.073 | 286.132.146 | 26.012.013 |
| 2010 | Histamin |  | 79 | 1.063 | 5.487 | 3.299 | 2.977 | 1.478 | 994 | 421 |  |  | 15.798 | 1.436 |
| 2010 | Indacaterol | 11.201.904 | 16.830.977 | 2.436.706 | 1.958.596 | 1.211.084 | 786.566 | 613.789 | 475.295 | 408.061 | 347.681 | 305.611 | 36.576.269 | 3.325.115 |
| 2010 | Meningokokken-konjugatimpfstoff | 2.929 | 7.009 | 10.470 | 10.705 | 12.898 | 16.570 | 9.675 | 6.668 | 1.526 | 267 | 4.736 | 83.451 | 7.586 |
| 2010 | Mifamurtid | 166 | 690 | 1.003 | 1.597 | 1.301 | 983 | 1.375 | 982 | 723 | 568 | 986 | 10.373 | 943 |
| 2010 | Pneumokokkenpolysaccharid-Konjugatimpfstoff |  |  |  |  |  |  |  |  |  |  |  | - | - |
| 2010 | Prucaloprid | 126.533 | 456.817 | 778.601 | 1.050.118 | 1.298.166 | 1.571.645 | 1.840.805 | 2.029.592 | 2.301.541 | 2.495.796 | 2.710.120 | 16.659.735 | 1.514.521 |
| 2010 | Roflumilast | 805.920 | 5.257.618 | 6.909.189 | 7.609.231 | 7.830.955 | 7.307.055 | 7.655.596 | 7.979.705 | 8.442.572 | 8.907.888 | 8.445.334 | 77.151.063 | 7.013.733 |
| 2010 | Silodosin | 445.319 | 1.805.178 | 2.293.448 | 3.036.798 | 3.707.294 | 4.099.632 | 4.591.232 | 4.950.677 | 5.641.749 | 6.293.277 | 6.342.591 | 43.207.195 | 3.927.927 |
| 2010 | Tapentadol | 261.361 | 2.761.256 | 4.635.218 | 5.996.854 | 7.578.733 | 8.862.689 | 10.237.297 | 11.619.947 | 13.099.295 | 14.610.094 | 15.108.778 | 94.771.523 | 8.615.593 |
| 2010 | Velaglucerase | 2.498 | 10.152 | 13.650 | 13.520 | 15.154 | 15.163 | 16.720 | 17.215 | 16.482 | 16.396 | 18.790 | 155.739 | 14.158 |
| 2010 | Vernakalant | 2 | 42 | 22 | 49 | 23 | 27 | 20 | 20 | 55 | 9 | 13 | 282 | 26 |
| 2011 | Abirateronacetat | 105.064 | 747.626 | 1.517.538 | 1.840.774 | 1.709.530 | 1.616.076 | 1.832.322 | 2.460.025 | 2.949.107 | 3.235.179 | 3.221.801 | 21.235.042 | 1.930.458 |
| 2011 | Apixaban | 1.489 | 19.607 | 3.513.632 | 19.666.977 | 51.828.059 | 95.069.802 | 146.184.421 | 201.245.826 | 255.187.973 | 309.292.178 | 353.976.661 | 1.435.986.624 | 130.544.239 |
| 2011 | Boceprevir | 62.752 | 295.633 | 160.355 | 32.670 | 429 |  |  |  |  |  |  | 551.839 | 50.167 |
| 2011 | Bromfenac | 518.401 | 2.821.989 | 738.320 | 20.545 | 741 |  |  |  |  |  |  | 4.099.996 | 372.727 |
| 2011 | Cabazitaxel | 2.001 | 128.297 | 139.589 | 140.347 | 129.905 | 151.492 | 147.199 | 172.165 | 179.792 | 213.123 | 87.256 | 1.491.166 | 135.561 |
| 2011 | Dexamfetamin | 328 | 103.303 | 195.969 | 172.053 | 208.560 | 313.498 | 475.720 | 631.508 | 730.419 | 783.663 | 854.669 | 4.469.690 | 406.335 |
| 2011 | Dexmedetomidin |  |  | 1 |  |  |  |  | 5 |  |  |  | 6 | 1 |
| 2011 | Epoprostenol | 8.118 | 46.937 | 66.802 | 46.792 | 11.915 | 9.761 | 11.377 | 5.001 |  |  |  | 206.702 | 18.791 |
| 2011 | Eribulin | 458 | 101.502 | 110.820 | 126.252 | 161.743 | 169.402 | 164.957 | 161.546 | 147.696 | 140.725 | 129.206 | 1.414.308 | 128.573 |
| 2011 | Fampridin | 371.994 | 2.889.535 | 3.570.006 | 3.989.575 | 4.260.393 | 4.356.975 | 4.464.771 | 4.680.784 | 4.791.213 | 5.015.719 | 5.241.596 | 43.632.562 | 3.966.597 |
| 2011 | Fingolimod | 496.353 | 1.341.373 | 2.152.243 | 2.991.456 | 3.512.804 | 3.928.164 | 4.222.349 | 4.657.571 | 4.850.961 | 4.915.351 | 4.738.425 | 37.807.051 | 3.437.005 |
| 2011 | Nabiximols | 141.884 | 420.757 | 501.527 | 632.697 | 779.531 | 933.802 | 1.392.454 | 1.794.263 | 2.116.127 | 2.410.995 | 2.510.763 | 13.634.799 | 1.239.527 |
| 2011 | Pirfenidon | 15.803 | 159.920 | 239.810 | 312.896 | 356.322 | 335.690 | 344.195 | 389.927 | 424.899 | 439.568 | 394.705 | 3.413.734 | 310.339 |
| 2011 | Pitavastatin | 1.818 | 1.864 | 91 | 102 |  | 324 | 1.171 | 2.552 | 4.053 | 6.080 | 6.905 | 24.960 | 2.269 |
| 2011 | Regadenoson |  | 196 | 425 | 159 | 9 | 31 | 28 | 19 | 53 | 374 | 492 | 1.787 | 162 |
| 2011 | Tafamidis | 433 | 8.371 | 13.484 | 16.200 | 16.042 | 18.635 | 23.446 | 25.449 | 57.139 | 188.190 | 378.532 | 745.920 | 67.811 |
| 2011 | Telaprevir | 66.265 | 304.892 | 131.189 | 15.341 | 490 | 272 |  |  |  |  |  | 518.450 | 47.132 |
| 2011 | Ticagrelor | 1.250.642 | 7.154.056 | 13.483.404 | 16.005.907 | 17.504.972 | 19.635.275 | 22.089.893 | 24.652.000 | 26.393.139 | 23.308.931 | 18.752.274 | 190.230.493 | 17.293.681 |

Tab. S1 Overview of drugs in defined daily doses (DDDs)

**References**

Ilan, Akker., Wolf, Sauter. (2022). Excessive pricing of pharmaceuticals in EU law: Balancing competition, innovation and regulation. 233-258. doi: 10.4337/9781800888708.00019

T., Dingermann. (2013). Das Arzneimittelmarktneuordnungsgesetz (AMNOG) und seine Folgen. Der Internist, 54(6):769-774. doi: 10.1007/S00108-013-3247-2

Garattini L, Finazzi B, Mannucci PM. Pharmaceutical pricing in Europe: time to take the right direction. Intern Emerg Med. 2022 Jun;17(4):945-948. doi: 10.1007/s11739-022-02960-8. Epub 2022 Mar 18. PMID: 35303264.

Germany Trade & Invest. (2024). *Pharmaceutical industry*. Retrieved October 18, 2024, from <https://www.gtai.de/en/invest/industries/healthcare-market-germany/pharmaceutical-industry#toc-anchor--1>

Gerd, Glaeske. (2012). The Dilemma Between Efficacy as Defined by Regulatory Bodies and Effectiveness in Clinical Practice. Deutsches Arzteblatt International, 109(7):115-116. doi: 10.3238/ARZTEBL.2012.0115

Guangxu, Jin., Stephen, T., C., Wong. (2014). Toward better drug repositioning: Prioritizing and integrating existing methods into efficient pipelines. Drug Discovery Today, 19(5):637-644. doi: 10.1016/J.DRUDIS.2013.11.005

Haserück A, Lau T, Osterloh F (2022) Preise steigen schneller als der Nutzen. Deutsches Ärzteblatt 119, 48. https:// www. aerzt eblatt. de/pdf/ 119/ 48/ a2128. pdf. Accessed 23rd October 2024

Valeska, Hofbauer-Milan., Stefan, Fetzer., Christian, Hagist. (2023). How to Predict Drug Expenditure: A Markov Model Approach with Risk Classes. PharmacoEconomics, 41(5):561-572. doi: 10.1007/s40273-023-01240-3

Klaus, Kaier., Stefan, Fetzer. (2015). Das Arzneimittelmarktneuordnungsgesetz (AMNOG) aus ökonomischer Sicht. Bundesgesundheitsblatt-gesundheitsforschung-gesundheitsschutz, 58(3):291-297. doi: 10.1007/S00103-014-2116-Z

Katrin, Kleining., J., Laufenberg., Philip, Thrun., Dorothee, Ehlert., Jürgen, Wasem., Arne, Bartol. (2023). Ten years of German benefit assessment: price analysis for drugs with unproven additional benefit.. Health Economics, Policy and Law, 1-18. doi: 10.1017/s1744133123000117

Victoria, Desirée, Lauenroth., Tom, Stargardt. (2017). Pharmaceutical Pricing in Germany: How Is Value Determined within the Scope of AMNOG?. Value in Health, 20(7):927-935. doi: 10.1016/J.JVAL.2017.04.006

Ludwig W, Mühlbauer B, Seifert R (2022) Arzneiverordnungs-Report 2022. Springer, Berlin/Heidelberg

Ludwig W, Mühlbauer B, Seifert R (2021) Arzneiverordnungs-Report 2021. Springer, Berlin/Heidelberg

Steven, M., Paul., Daniel, S., Mytelka., Dunwiddie, Christopher, T., Charles, C., Persinger., Bernard, H., Munos., Stacy, R., Lindborg., Aaron, Leigh, Schacht. (2010). How to improve R&D productivity: the pharmaceutical industry's grand challenge. Nature Reviews Drug Discovery, 9(3):203-214. doi: 10.1038/NRD3078

Barbara, Mintzes., Ellen, Clarissa, Reynolds., Priya, Bahri., Lucy, T, Perry., Alice, Bhasale., Richard, L., Morrow., Colin, R., Dormuth. (2022). How do safety warnings on medicines affect prescribing?. Expert Opinion on Drug Safety, 21(10):1269-1273. doi: 10.1080/14740338.2022.2134342

Domenico, Motola., Fabrizio, De, Ponti., Elisabetta, Poluzzi., Nello, Martini., Pasqualino, Rossi., Maria, Chiara, Silvani., Alberto, Vaccheri., Nicola, Montanaro. (2006). An update on the first decade of the European centralized procedure: how many innovative drugs?. British Journal of Clinical Pharmacology, 62(5):610-616. doi: 10.1111/J.1365-2125.2006.02700.X

Mathias, Møllebæk., Susanne, Kaae., Marie, L., De, Bruin., Torbjörn, Callréus., Sukhwinder, Jossan., Christine, E., Hallgreen. (2019). The effectiveness of direct to healthcare professional communication - A systematic review of communication factor studies.. Research in Social & Administrative Pharmacy, 15(5):475-482. doi: 10.1016/J.SAPHARM.2018.06.015

Paffrath D, Ludwig W, Klauber J, Schwabe U (2017) Arzneiverordnungs-Report 2017: aktuelle Daten, Kosten, Trends und Kommentare. Springer, Berlin/Heidelberg

Frank, Peinemann., Alexander, Labeit. (2019). Varying results of early benefit assessment of newly approved pharmaceutical drugs in Germany from 2011 to 2017: A study based on federal joint committee data.. Journal of Evidence-based Medicine, 12(1):9-15. doi: 10.1111/JEBM.12340

Sigrid, Piening., K., Reber., Jaap, E., Wieringa., Sabine, M., J., M., Straus., de, Pieter, Graeff., Flora, M., Haaijer-Ruskamp., Peter, G., M., Mol. (2012). Impact of Safety‐Related Regulatory Action on Drug Use in Ambulatory Care in the Netherlands. Clinical Pharmacology & Therapeutics, 91(5):838-845. doi: 10.1038/CLPT.2011.308

Alexander, Schuhmacher., Markus, Hinder., Alexander, von, Stegmann, und, Stein., Dominik, Hartl., Oliver, Gassmann. (2023). Analysis of pharma R&D productivity - a new perspective needed.. Drug Discovery Today, 103726-103726. doi: 10.1016/j.drudis.2023.103726

Schwabe U, Ludwig W (2020) Arzneiverordnungs-Report 2020. Springer, Berlin/Heidelberg

Schwabe U, Paffrath D (2005) Arzneiverordnungs-Report 2005: Aktuelle Daten, Kosten, Trends und Kommentare. Springer, Berlin/Heidelberg

Schwabe U, Paffrath D (2006) Arzneiverordnungs-Report 2006: Aktuelle Daten, Kosten, Trends und Kommentare. Springer Berlin/Heidelberg

Schwabe U, Paffrath D (2007) Arzneiverordnungs-Report 2007: Aktuelle Daten, Kosten, Trends und Kommentare. Springer Berlin/Heidelberg

Schwabe U, Paffrath D (2008) Arzneiverordnungs-Report 2008: Aktuelle Daten, Kosten, Trends und Kommentare. Springer Berlin/Heidelberg

Schwabe U, Paffrath D (2009) Arzneiverordnungs-Report 2009: Aktuelle Daten, Kosten, Trends und Kommentare. Springer Berlin/Heidelberg

Schwabe U, Paffrath D (2010) Arzneiverordnungs-Report 2010: Aktuelle Daten, Kosten, Trends und Kommentare. Springer Berlin/Heidelberg

Schwabe U, Paffrath D (2011) Arzneiverordnungs-Report 2011: Aktuelle Daten, Kosten, Trends und Kommentare. Springer Berlin/Heidelberg

Schwabe U, Paffrath D (2012) Arzneiverordnungs-Report 2012: Aktuelle Daten, Kosten, Trends und Kommentare. Springer Berlin/Heidelberg

Schwabe U, Paffrath D (2013) Arzneiverordnungs-Report 2013: Aktuelle Daten, Kosten, Trends und Kommentare. Springer Berlin/Heidelberg

Schwabe U, Paffrath D (2014) Arzneiverordnungs-Report 2014: Aktuelle Daten, Kosten, Trends und Kommentare. Springer Berlin/Heidelberg

Schwabe U, Paffrath D (2015) Arzneiverordnungs-Report 2015: Aktuelle Daten, Kosten, Trends und Kommentare. Springer Berlin/Heidelberg

Schwabe U, Paffrath D (2016) Arzneiverordnungs-Report 2016: Aktuelle Daten, Kosten, Trends und Kommentare. Springer Berlin/Heidelberg

Schwabe U, Paffrath D, Ludwig W, Klauber J (2018) Arzneiverordnungs-Report 2018: aktuelle Daten, Kosten, Trends und Kommentare. Springer, Berlin/Heidelberg

Schwabe U, Paffrath D, Ludwig W, Klauber J (2019) Arzneiverordnungs-Report 2019: aktuelle Daten, Kosten, Trends und Kommentare. Springer, Berlin/Heidelberg

Carsten, Schwenke., Susanne, Schwenke. (2018). Die frühe Nutzenbewertung von Arzneimitteln gemäß § 35a SGB V. 113-141. doi: 10.1007/978-3-658-15987-0_6

Ned, Stafford. (2014). Only 3 in 20 new drugs approved in Germany in 2011 were an improvement, report says. BMJ, 348 doi: 10.1136/BMJ.G2657

Ned, Stafford. (2015). German drug companies are criticised for not focusing on useful therapeutic areas. BMJ, 351 doi: 10.1136/BMJ.H4938

Beate, Wieseler., Natalie, McGauran., Thomas, M., Kaiser. (2019). New drugs: where did we go wrong and what can we do better?. BMJ, 366(12):727-731. doi: 10.1136/BMJ.L4340
